# A Ca^2+^/CaM-regulated transcriptional switch modulates stomatal development in response to water deficit

**DOI:** 10.1038/s41598-019-47529-2

**Published:** 2019-08-22

**Authors:** Chan Yul Yoo, Noel Mano, Aliza Finkler, Hua Weng, Irene S. Day, Anireddy S. N. Reddy, B. W. Poovaiah, Hillel Fromm, Paul M. Hasegawa, Michael V. Mickelbart

**Affiliations:** 1 Department of Horticulture and Landscape Architecture, Purdue University, West Lafayette, IN 47907 USA; 2 Department of Botany and Plant Pathology, Purdue University, West Lafayette, IN 47907 USA; 3 School of Plant Sciences and Food Security, Faculty of Life Sciences, Tel Aviv University, Tel Aviv, 6997801 Israel; 4 Department of Biology and Program in Cell and Molecular Biology, Colorado State University, Fort Collins, CO 80523 USA; 5 Department of Horticulture, Washington State University, Pullman, WA 99164 USA; 6 Present Address: Department of Botany and Plant Sciences, Institute of Integrative Genome Biology, University of California Riverside, Riverside, CA 92521 USA

**Keywords:** Plant signalling, Abiotic

## Abstract

Calcium (Ca^2+^) signals are decoded by the Ca^2+^-sensor protein calmodulin (CaM) and are transduced to Ca^2+^/CaM-binding transcription factors to directly regulate gene expression necessary for acclimation responses in plants. The molecular mechanisms of Ca^2+^/CaM signal transduction processes and their functional significance remains enigmatic. Here we report a novel Ca^2+^/CaM signal transduction mechanism that allosterically regulates DNA-binding activity of GT2-LIKE 1 (GTL1), a transrepressor of *STOMATAL DENSITY AND DISTRIBUTION 1* (*SDD1*), to repress stomatal development in response to water stress. We demonstrated that Ca^2+^/CaM interaction with the 2^nd^ helix of the GTL1 N-terminal trihelix DNA-binding domain (GTL1N) destabilizes a hydrophobic core of GTL1N and allosterically inhibits 3^rd^ helix docking to the *SDD1* promoter, leading to osmotic stress-induced Ca^2+^/CaM-dependent activation (de-repression) of *SDD1* expression. This resulted in GTL1-dependent repression of stomatal development in response to water-deficit stress. Together, our results demonstrate that a Ca^2+^/CaM-regulated transcriptional switch on a trihelix transrepressor directly transduces osmotic stress to repress stomatal development to improve plant water-use efficiency as an acclimation response.

## Introduction

Plants sense and respond to external stimuli to acclimate and adapt to diverse environmental niches. In response to water deficit, plants limit transpirational water loss via reduced stomatal aperture as a rapid plant response^1^. As a longer-term response, plants may repress stomatal development, the result of which is reduced water loss to improve water-use efficiency (WUE) and/or drought tolerance^2,3^. Hyperosmotic stress induces signaling pathways that activate or repress genes necessary for water-deficit acclimation responses^4^ and transcriptional regulation is a key regulatory mechanism that governs stomatal development^5^. The abundance of SPEECHLESS, the master transcriptional regulator of stomatal cell identity, is decreased by osmotic stress via a mitogen-activated protein kinase (MAPK) cascade^6,7^. However, the earliest signal transduction mechanism from sensing water-deficit to the direct regulation of gene expression responsible for repression of stomatal development remains enigmatic.

Calcium ions (Ca^2+^) are versatile second messengers regulating plant growth and development. Environmental stimuli, and specifically hyperosmotic stress, induce rapid and transient changes in cytosolic and nuclear Ca^2+^ concentrations that differ in amplitude and duration (Ca^2+^ signatures)^8–12^. These stimuli-specific Ca^2+^ changes are decoded by Ca^2+^ sensor proteins, including the ubiquitous Ca^2+^-binding protein calmodulin (CaM). Ca^2+^ binding to CaM induces conformational change from a closed to an open state, facilitating hydrophobic and electrostatic interactions with basic amphipathic helices of target peptides to activate or repress the molecular activity of target proteins^9–11^. Among various CaM-binding targets, Ca^2+^/CaM-binding transcription factors, such as the calmodulin-binding transcriptional activator (CAMTA), myeloblastosis (MYB), and basic helix-loop-helix (bHLH) families, transduce signals to activate or repress gene expression necessary for inducing cellular responses^12–20^. However, the molecular and structural mechanisms of signal transduction by which Ca^2+^/CaM regulates transcriptional activity or DNA-binding activity of transcription factors are not fully understood.

Trihelix transcription factors are characterized by trihelical (α1-loop-α2-loop-α3) DNA-binding domains that specifically bind to the GT motif^21,22^. GT1 and GT2 sub-families are distinguished by the presence of one or two trihelical DNA-binding domains, respectively. The solution structure of the *Arabidopsis* GT-1 DNA-binding domain by NMR spectroscopy revealed that the trihelical tertiary structure is stabilized by hydrophobic residues forming a hydrophobic core that allows α3 docking to the GT1 box in target gene promoters^23^. GT2 factors have been implicated in developmental, light, and abiotic stress responses in *Arabidopsis*, poplar, soybean, and wheat^24–30^. We previously reported that the *Arabidopsis* GT2-LIKE 1 (GTL1) is a transrepressor of *STOMATAL DENSITY AND DISTRIBUTION 1* (*SDD1*) that negatively regulates stomatal development and transpiration^27,28^. Ca^2+^/CaM-binding affinity has been demonstrated in AtGT2L and PtaGTL1^26,31^. However, Ca^2+^/CaM signal transduction mechanisms of GTL1 and their biological implications are not known. Here, we show that Ca^2+^/CaM directly binds to the N-terminal DNA-binding domain of GTL1 and allosterically inhibits DNA-binding activity by disturbing the hydrophobic core. Hyperosmotic stress-induced Ca^2+^/CaM allosteric control on the GTL1 transrepressor leads to *SDD1* derepression, both by inhibiting binding to and promoting release from the *SDD1* promoter, leading to repression of stomatal development in response to water-deficit stress. We propose that this allosteric control of the GTL1 transcription factor by Ca^2+^/CaM is a transcriptional switch to modulate stomatal development, thereby conserving plant water loss as a long-term developmental adaptation during water stress.

## Results

### Ca^2+^/CaM binds to the GTL1 N-terminal trihelical DNA-binding domain

**Figure 1:**
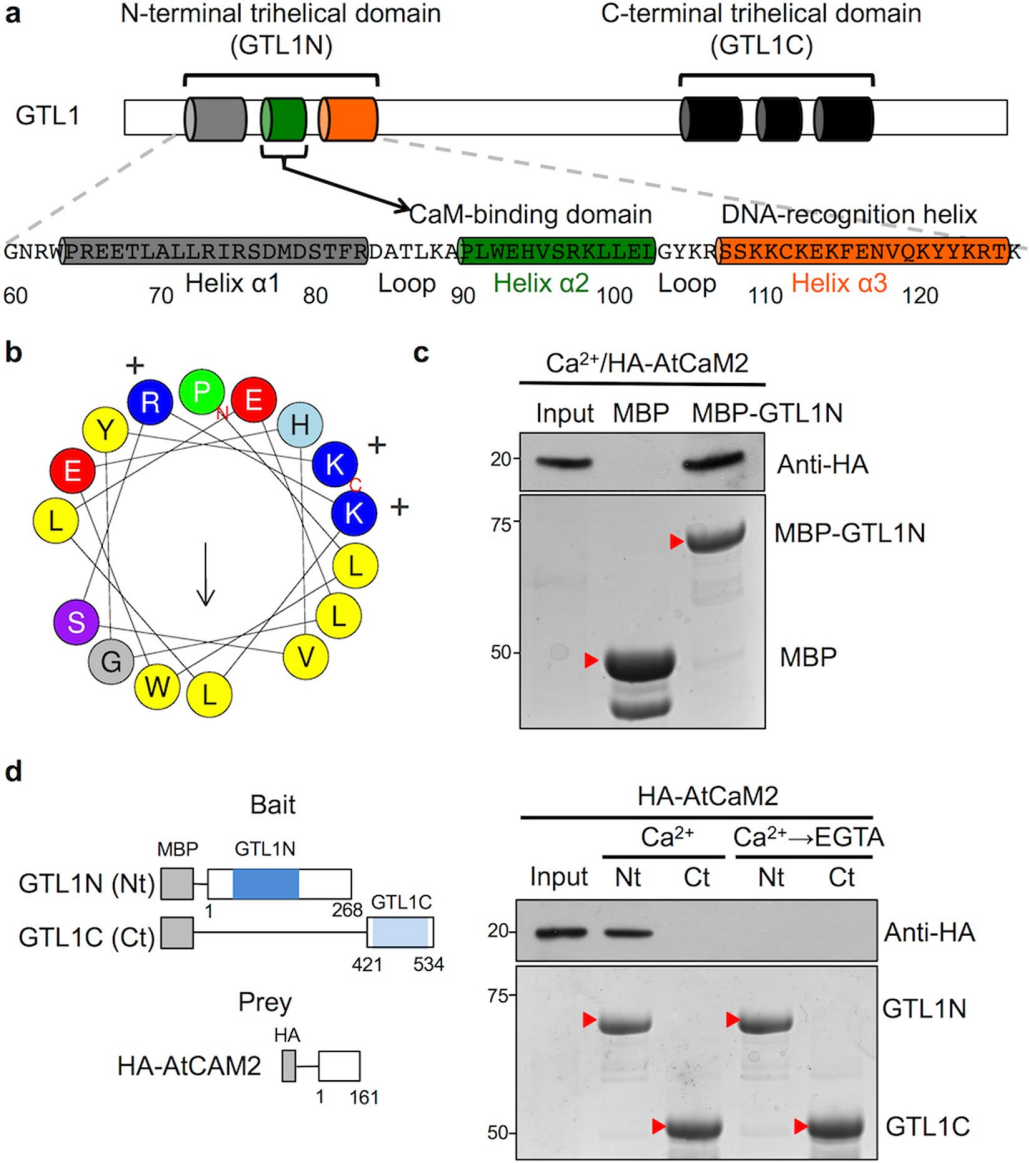
CaM binds to the N-terminal DNA-binding domain of GTL1 in a Ca^2+^-dependent manner. (**a**) Schematic illustration of GTL1 domain topology with N- and C-terminal trihelical domains. The primary and secondary structure of GTL1N (residue number 60~126) consists of three α-helices (α1 – grey, α2 – green, and α3 – orange) with α2 containing a predicted CaM-binding domain. (**b**) The helical wheel projection of the α2 helix shows a canonical amphipathic helix with hydrophobic (yellow) and hydrophilic (basic – blue and acidic – red) residues. (**c**) GTL1N interaction with Ca^2+^/CaM was performed by *in vitro* pull-down assay using *Escherichia coli* expressed MBP-GTL1N or MBP to pull down *in vitro-*translated HA-AtCaM2 that was detected by immunoblots using anti-HA antibodies (upper panel). Immobilized MBP and MBP-GTL1N fusion proteins are shown in the Coomassie blue-stained SDS-PAGE gel (lower panel). (**d**) Schematic illustration of the bait and prey proteins used in the pull-down assays. Ca^2+^-dependent CaM interaction with GTL1N or GTL1C were performed by pull-down assay using HA-AtCaM2. Bound and 10% of input HA-AtCaM2 fractions were detected by immunoblot using anti-HA antibodies. The MBP-GTL1N (Nt) and MBP-GTL1C (Ct) fusion protein bands are indicated by red arrows. Full-length blots and gels for c and d are presented in Supplementary Fig. S10.

We hypothesized that Ca^2+^/CaM directly binds to AtGTL1, a negative regulator of stomatal development, to mediate water-deficit stress acclimation. The domain structure of GTL1 includes two trihelical DNA-binding domains in the N- and C-termini (Fig. 1a). A prediction algorithm identifies the α2 helix within the N-terminal DNA-binding domain (GTL1N) as a putative CaM-binding domain, but not within the C-terminal DNA-binding domain (GTL1C) (Supplementary Fig. S1)^32^. The helical projection of α2 indicates a canonical structure of a CaM-binding domain, which is a basic amphipathic helix with hydrophobic residues on one side and positively-charged basic residues on the other side (Fig. 1b). An *in vitro* pull-down assay using HA-*Arabidopsis* CaM2 (AtCaM2) showed that HA-AtCaM2 directly interacted with the GTL1N fused with maltose-binding protein (MBP) in the presence of Ca^2+^, but not with the MBP alone (Fig. 1c). The interaction between Ca^2+^/CaM and GTL1N was abolished by EGTA treatment, a chelator of Ca^2+^, indicating that Ca^2+^ is required for the interaction between CaM and GTL1N (Fig. 1d). In addition, the Ca^2+^-dependent CaM interaction only occurred with GTL1N, but not with GTL1C, indicating that the CaM-binding property is specific to the N-terminal trihelical DNA-binding domain (Fig. 1d). An *in vitro* CaM overlay assay using AtCaM2-conjugated with horseradish peroxidase (HRP) further confirms that the interaction of GTL1N and AtCAM2 occurred only in the presence of Ca^2+^ (Supplementary Fig. S2). Together, these results indicate that GTL1 is a Ca^2+^/CaM-binding protein and Ca^2+^/CaM directly interacts with the N-terminal DNA-binding domain, presumably via the α2 helix.

**Figure 2:**
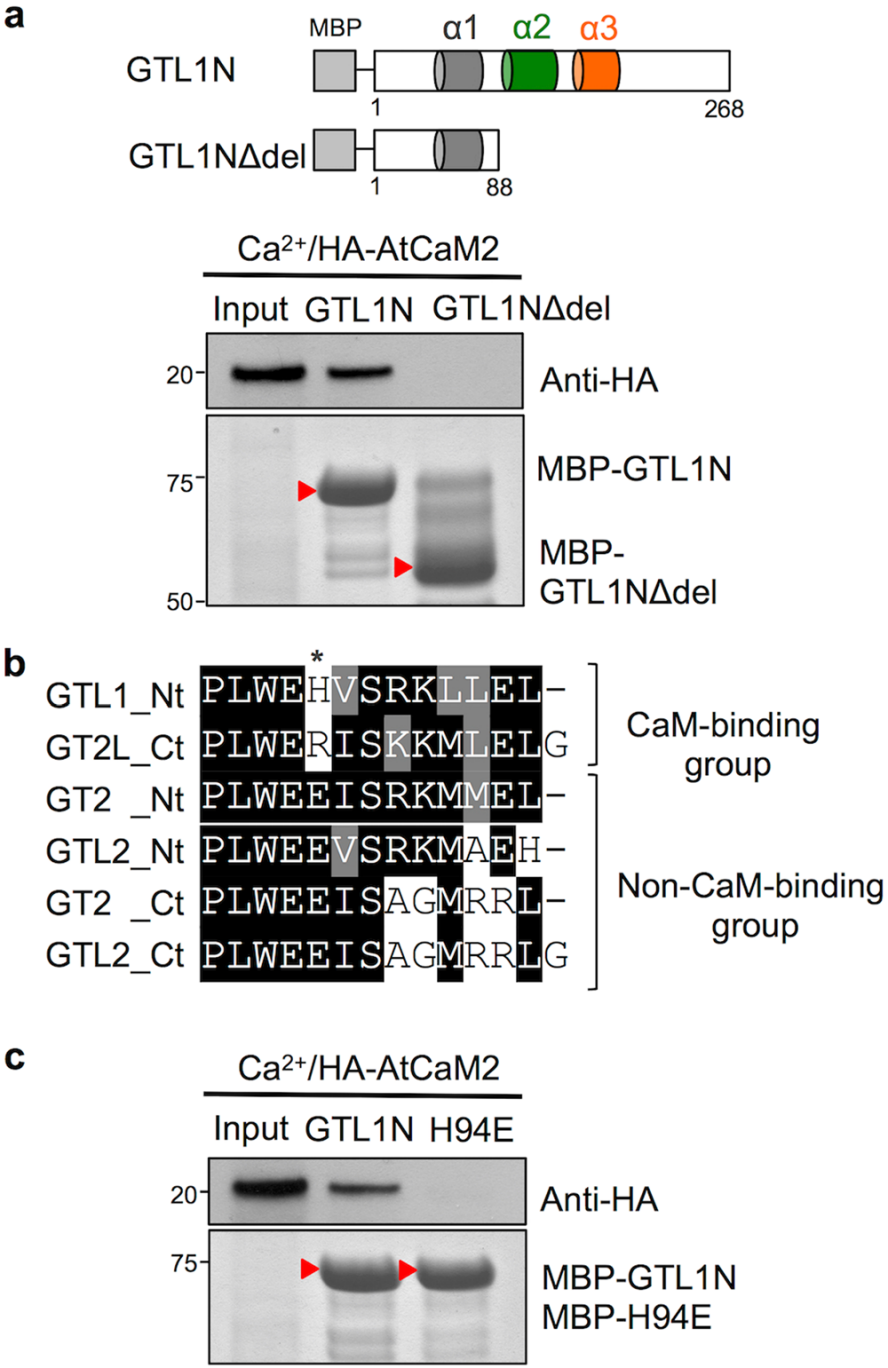
Ca^2+^/CaM binds to the α2 helix of GTL1N. (**a**) Schematic illustration of two protein fragments (GTL1N and GTL1N∆del) fused to maltose-binding protein (MBP). *In vitro* pull-down assay was performed using MBP-fusion proteins with Ca^2+^/HA-AtCaM2. Bound and 10% of input HA-AtCaM2 fractions were detected by immunoblot using anti-HA antibodies. The MBP-GTL1N and MBP- GTL1N∆del fusion protein bands are indicated by red arrows. (**b**) Sequence alignment of the α2 helix of the N-terminal (Nt) or C-terminal (Ct) trihelical domain from GT2 family proteins, including GTL1. AtGTL1_Nt and AtGT2L_Ct have CaM-binding activity. *Indicates the residue that distinguishes CaM-binding and non-CaM-binding groups. (**c**) An *in vitro* pull-down assay was performed using MBP-GTL1N or MBP-GTL1N[H94E] fusion proteins with Ca^2+^/HA-AtCaM2. Bound and 10% of input HA-AtCaM2 fractions were detected by immunoblot using anti-HA antibodies. The MBP-GTL1N and MBP-GTL1N[H94E] fusion protein bands are indicated by red arrows. Full-length gels for a and c are presented in Supplementary Fig. S10.

To confirm that the α2 helix contains a CaM-binding domain, the GTL1N∆del fragment (without α2 and α3 helices) was tested for interaction with Ca^2+^/CaM. GTL1N∆del completely lost binding affinity to Ca^2+^/CaM (Fig. 2a), confirming the *in silico* prediction of α2 as a CaM-binding domain. To identify the critical residue important for CaM binding, the amino acid sequences of the α2 helix of the N- and C-terminal DNA-binding domains from CaM-binding and non-CaM binding groups of GT2 family proteins were aligned (Fig. 2b). The H94 residue in α2 of GTL1 has a similar basic property to the arginine (R501) in α2 of AtGT2L, another CaM-binding GT2 family protein^31^. The alternative residue for H94 of GTL1 and R501 of AtGT2L is glutamic acid (E) in the non-CaM binding group, suggesting that the H94 residue may determine the CaM-binding property of the α2 helix. To test this hypothesis, we introduced a site-directed mutation by substituting histidine for glutamic acid (H94E) in GTL1N and tested for CaM-binding activity. An H94E substitution resulted in the loss of CaM binding (Fig. 2c), presumably due to electrostatic repulsion with conserved E residues of CaM^9,11^. These results indicate that Ca^2+^/CaM binds to GTL1N α2, and that H94 is a critical residue for interaction with Ca^2+^/CaM.

### The GTL1 N-terminal trihelical DNA-binding domain requires hydrophobic core formation for its DNA-binding activity

**Figure 3:**
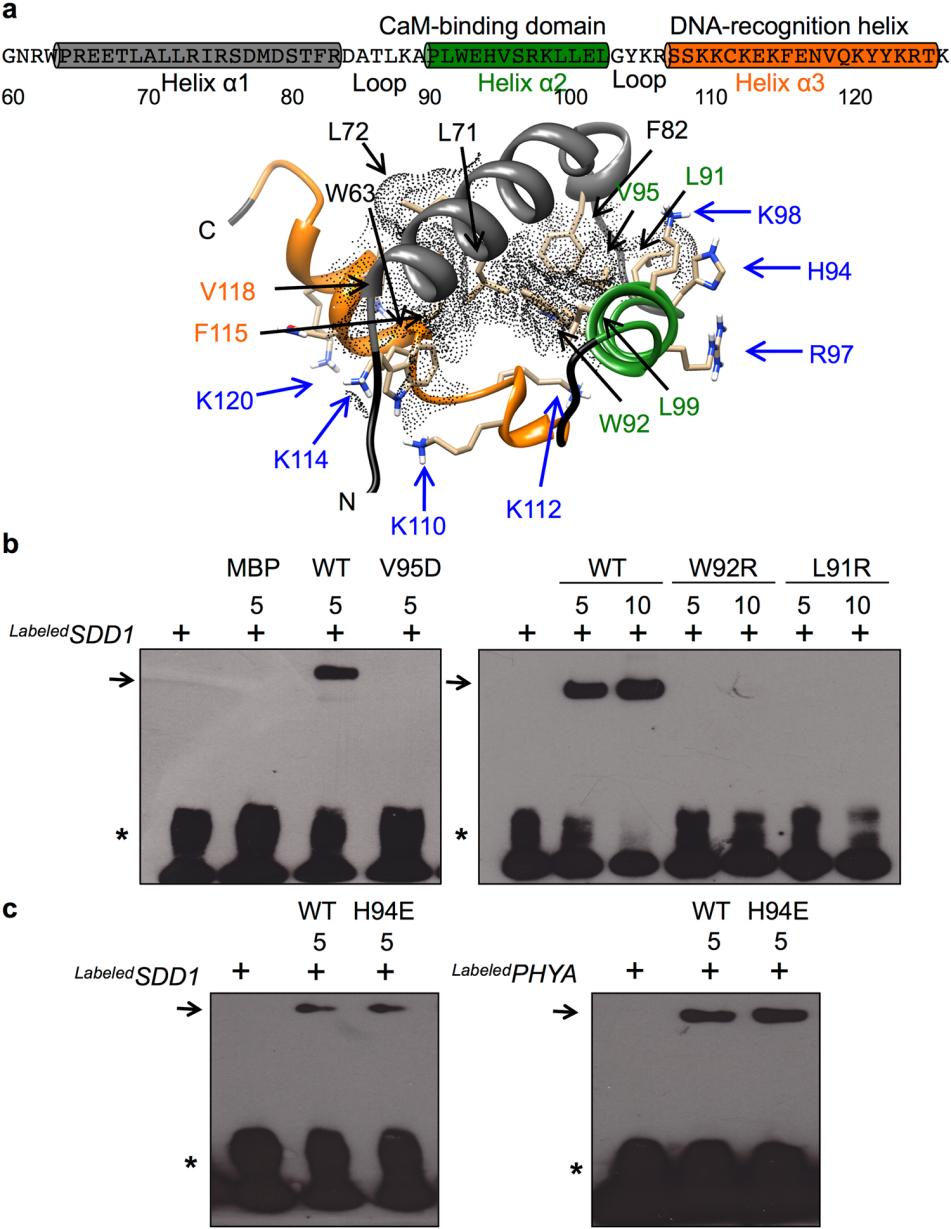
The GTL1 N-terminal DNA-binding domain forms a trihelical tertiary structure that is stabilized by hydrophobic residues. (**a**) Schematic illustration of GTL1 N-terminal trihelical domain. The primary and secondary structure of GTL1N (Residue Number 60–126) consists of three helices with α2 (green) and α3 (orange) helices containing CaM-binding and DNA-recognition sequence motifs, respectively. The three-dimensional GTL1N structure is predicted to be stabilized by a hydrophobic core (grey dot area) that is formed by hydrophobic residues shown in black, green, and orange distributed in the three helices. Blue residues indicate charged residues necessary for DNA recognition (α3) and CaM binding (α2). (**b**) DNA-binding activity of recombinant proteins (5 or 10 μg) of MBP, GTL1N (WT), or GTL1N mutations ([V95D], [W92R], and [L91R]) with biotin-labeled *SDD1* promoter fragments was performed by electrophoretic mobility shift assay (EMSA). (**c**) GTL1N (WT) or H94E proteins were used to determine binding activity with a biotin-labeled *SDD1* promoter fragment (left panel) or rice *PHYA* promoter fragment (right panel). Free promoter and protein-promoter complexes are indicated by an asterisk and arrows, respectively.

Ca^2+^/CaM binding to the α2 helix within the GTL1 N-terminal trihelical DNA-binding domain suggests a potential link between Ca^2+^/CaM binding and the regulation of DNA-binding activity. To understand the functional characteristics of the trihelical domain, the structure of GTL1N was constructed by comparative homology modeling with the NMR structure of the AtGT-1 trihelical DNA-binding domain as a template^23^. The primary and secondary structural comparison indicates that the key residues required for hydrophobic core formation in GT-1 are conserved in the GTL1N trihelical DNA-binding domain with 35.94% sequence identity (Supplementary Fig. S3a), which is above 30%, a good standard for accuracy of homology modeling^33^. The modeled GTL1N trihelical domain indicates that GTL1N α1 (residues 65–83) and α2 (residues 90–102) are oblique to each other, and that α3 (residues 107–125) is perpendicular to α1 (Fig. 3a). Ten residues (N terminus-W63; α1-L71, L72, and F82; α2-L91, W92, V95, and L99; and α3-F115 and V118) form a hydrophobic core to stabilize the GTL1N tertiary structure, which is conserved with hydrophobic residues important for the hydrophobic core of GT-1 (Fig. 3a and Supplementary Fig. S3b). Charged residues on the α3 helix, important for electrostatic interactions with the GT box *cis-*element, are also conserved between GT-1 and GTL1 (Supplementary Fig. S3c), suggesting that the functional structure of the trihelix DNA-binding domain is conserved between GT-1 and GTL1.

The GTL1N tertiary structure allows the α3 helix to interact with the major groove of the GT-3 box in the predicted docking structure between GTL1N and the *SDD1* promoter (5′-gcttGGTAAAactt-3′) (Supplementary Fig. S4). The predicted model suggests that GTL1N-*SDD1* promoter interactions occur via hydrogen bonds between α3 K109 (hydrogen donor) and the first guanine (Gua1) of the GT3 box (hydrogen acceptor), and α3 E116 (hydrogen acceptor) and the fourth adenine (Ade4) of the GT3 box (hydrogen donor) (Supplementary Fig. S4b). Other residues W63, α2-W92, and α3-K110, K112, K114, N117, Q119, and K120 interact with the negatively charged phosphate backbone of the GT3 box (Supplementary Fig. S4c), which stabilizes the GTL1N-*SDD1* promoter interaction. Based on the docking model of GTL1N-*SDD1* promoter, we hypothesize that a hydrophobic core in the trihelical GTL1N DNA-binding domain is essential for its DNA-binding activity. To validate the importance of the hydrophobic core in tertiary structure for DNA-binding activity, hydrophobic residues (L91, W92, and V95) were substituted to acidic or basic amino acids to disrupt the formation of a hydrophobic core. Consistent with the homology modeling and docking prediction, site-directed mutations (L91R, W92R, and V95D) of hydrophobic residues to charged residues results in the complete loss of binding to the *SDD1* promoter fragment (Fig. 3b). These data support the model that the hydrophobic core formed by the hydrophobic residues in the GTL1N trihelical structure is required for native folding and its function as a DNA-binding transcription factor. However, the H94E mutation in the H94 residue required for Ca^2+^/CaM interaction did not result in the loss of DNA binding to the *SDD1* promoter or rice *PHYA* promoter (Fig. 3c), suggesting that the α2 helix is not directly involved with DNA-recognition. Together, this result further supports the notion that the hydrophobic core formation results in a protein topology for surface exposure of the α2 (H94, R97, and K98) and α3 (K110, K112, K114, and K120) basic residues that are necessary for CaM and DNA interactions, respectively.

### Ca^2+^/CaM binding to α2 of GTL1N inhibits its association with and facilitates dissociation from the *SDD1* promoter

**Figure 4:**
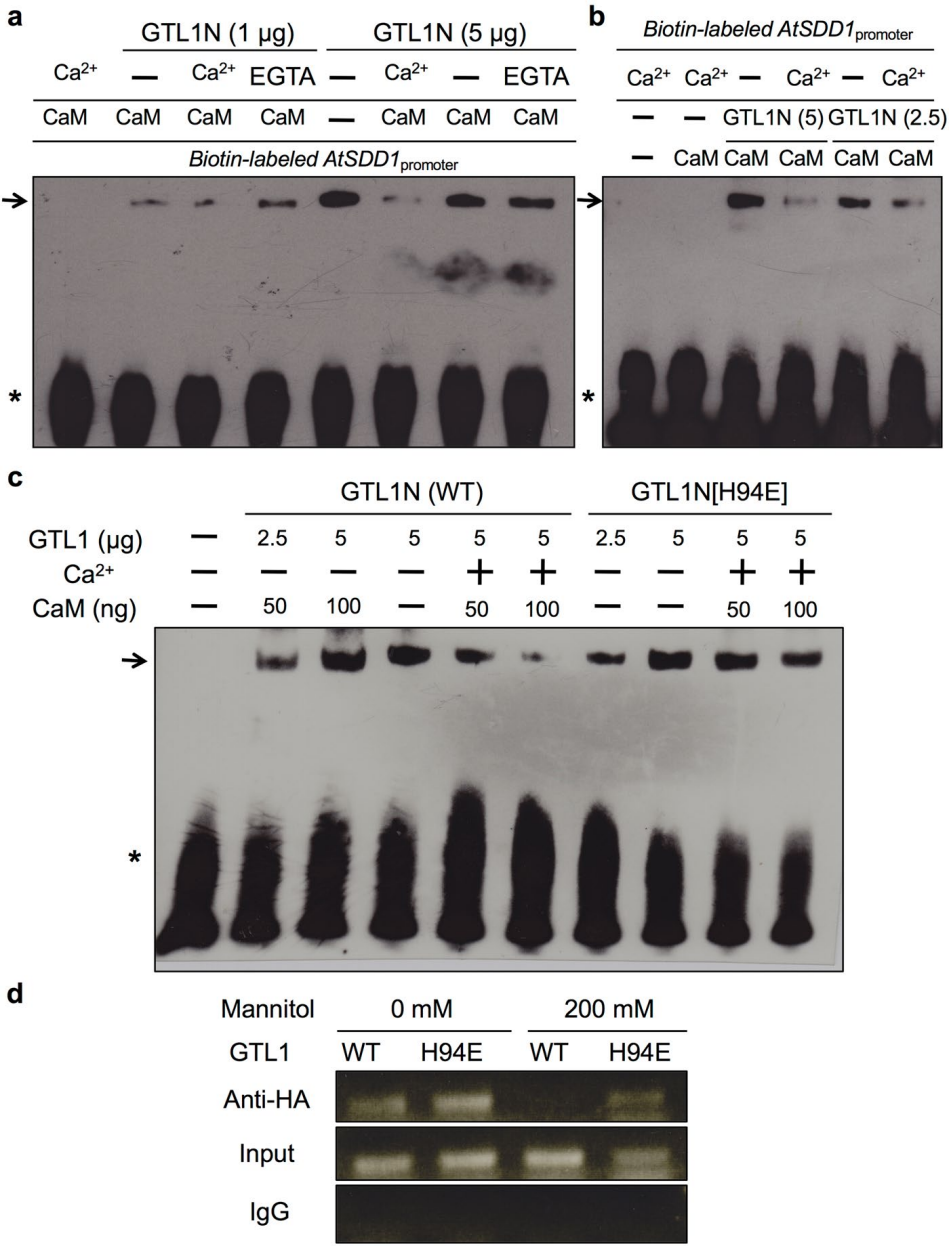
Hyperosmotic stress-induced Ca^2+^/CaM binding to the α2 helix inhibits DNA-binding activity of GTL1 to the *SDD1* promoter. (**a–c**) EMSA was performed using GTL1N or GTL1N[H94E], and a biotin-labeled *SDD1* promoter fragment that includes the GT3 box. (**a**) GTL1N (1 or 5 mg) was pre-incubated with human CaM (100 ng) without or with 2 mM CaCl_2_ or 10 mM EGTA, and then incubated with the biotin-labeled *SDD1* promoter fragment (250 ng). (**b**) GTL1N (2.5 or 5 mg) was pre-incubated with the *SDD1* promoter fragment without or with 2 mM CaCl_2_, and then with human CaM (100 ng). (**c**) GTL1N or GTL1N[H94E] was pre-incubated with the *SDD1* promoter fragment without or with 2 mM CaCl_2_ and then with human CaM (50 or 100 ng). (**d**) ChIP assays using anti-HA antibody were performed using protoplasts isolated from *gtl1-4* plants, transformed with *HA-GTL1* or *HA-GTL1*[*H94E*], and incubated with 0 or 200 mM mannitol for 1 h. Input is the total isolated chromatin before immunoprecipitation. Mouse IgG was the negative control for immunoprecipitation. The *SDD1* promoter region including the GT3 box was amplified by *SDD1* promoter-specific primers.

Ca^2+^/CaM binding to the α2 helix within the GTL1 N-terminal DNA-binding domain prompted us to postulate that Ca^2+^/CaM binding may allosterically inhibit GTL1N DNA binding to the *SDD1* promoter. This hypothesis is based on the typical Ca^2+^/CaM-target peptide structure requiring hydrophobic residues^9,34^, which are important to form the hydrophobic core of GTL1N (Fig. 3a). We first tested this hypothesis with an *in vitro* electrophoretic mobility shift assay (EMSA) that was previously used to show GTL1 DNA binding to the *SDD1* promoter^27^. GTL1N was able to interact with the *SDD1* promoter fragment in the presence of CaM or CaM with EGTA (Fig. 4a). However, addition of Ca^2+^ substantially reduced the binding affinity with *SDD1* (Fig. 4a). Interestingly, when GTL1N was pre-incubated with the *SDD1* promoter fragment, the addition of CaM in the presence of Ca^2+^ was also able to dissociate GTL1N from the *SDD1* promoter fragment (Fig. 4b). This result indicates that Ca^2+^/CaM is able to dissociate GTL1N from the *SDD1* promoter. To further confirm the effect of Ca^2+^/CaM on GTL1N DNA-binding activity, the EMSA was performed using GTL1N[H94E] that is unable to bind Ca^2+^/CaM (Fig. 2c). The addition of CaM in the presence of Ca^2+^ was able to dissociate the GTL1N-*SDD1* promoter complex, but not the GTL1N[H94E]-*SDD1* promoter complex (Fig. 4c). These *in vitro* assays indicate that Ca^2+^/CaM binding to the α2 helix of GTL1N inhibits association and facilitates dissociation from the *SDD1* promoter.

To recapitulate these results *in vivo* using full-length GTL1 protein, a chromatin-immunoprecipitation (ChIP) assay was performed in *gtl1-4* protoplasts expressing wild-type HA-GTL1 or mutant HA-GTL1[H94E]. Both GTL1 and GTL1[H94E] proteins associated with the *SDD1* promoter region that contains the GT3 box, indicating again that the GTL1[H94E] protein is functional (Fig. 4d). To activate *in planta* Ca^2+^ signaling, 200 mM mannitol was used to induce hyperosmotic stress, which has been reported to induce cytosolic and nuclear Ca^2+^ transients in tobacco and *Arabidopsis*^35–38^. Mannitol reduced GTL1 association with the *SDD1* promoter, but did not substantially reduce GTL1[H94E] association with the *SDD1* promoter (Fig. 4d). Together, both *in vitro* and *in vivo* results indicate that hyperosmotic stress-induced Ca^2+^/CaM attenuates GTL1 docking to the *SDD1* promoter.

### Hyperosmotic stress induces *SDD1* expression through Ca^2+^/CaM-dependent inhibition of the GTL1 transrepressor

**Figure 5:**
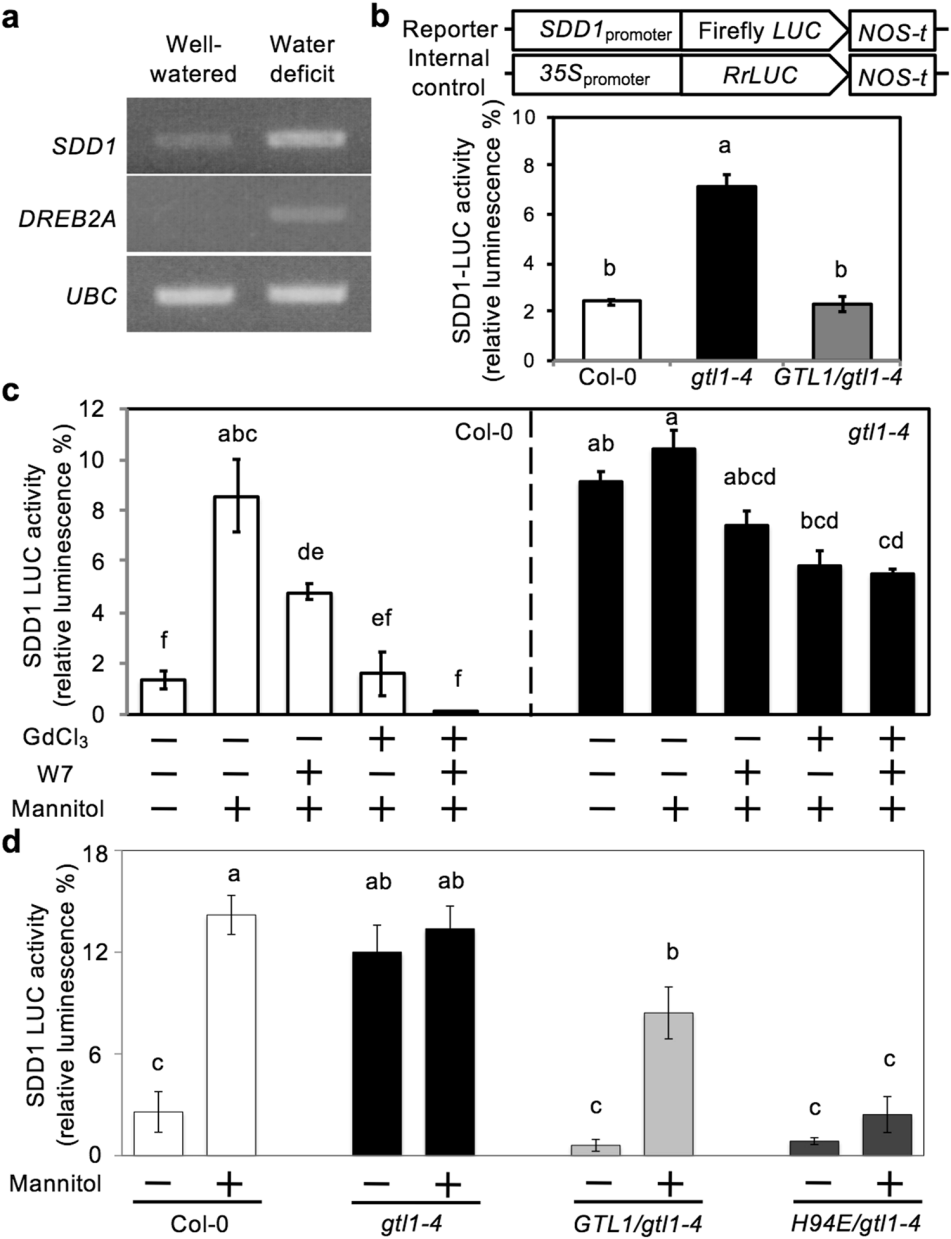
Hyperosmotic stress induces *SDD1* expression through Ca^2+^/CaM-dependent inhibition of the GTL1 transrepressor. (**a**) Transcript abundance of *SDD1*, *DREB2A*, and *UBC* was determined in total RNA extracted from 5-week-old Col-0 plants that were well-watered or water-deficit stressed. (**b**) Schematic diagram of the reporter and internal control constructs. The reporter construct included the 2 kb *SDD1*_promoter_ fused with firefly luciferase (*LUC*) gene, and nopaline synthase termination signal (*NOS-t*). The internal control construct included the *35S*_promoter_, *Renilla reniformis LUC* (*RrLUC*), and *NOS-t*. SDD1-LUC activity relative to RrLUC activity (% of SDD1-LUC to RrLUC activity) was measured from protoplasts isolated from Col-0 (wild type), *gtl1-4*, and *gtl1-4* expressing *GTL1* (*GTL1/gtl1-4*). (**c**) Relative SDD1-LUC activities were determined in Col-0 and *gtl1-4* protoplasts that were incubated with or without 1 mM GdCl_3_, 600 mM W7, or GdCl_3_ and W7 combined prior to addition of mannitol to a final concentration of 200 mM. (**d**) SDD1- LUC activities of Col-0, *gtl1-4*, or *HA-GTL1* or *HA-GTL1*[*H94E*] protoplasts were determined after incubation with 0 or 200 mM mannitol for 1 h. All results shown are mean ± SEM (*n* = 3). Columns with the same letters above are not significantly different from each other based on Tukey’s Honestly Significant Difference (HSD) test (*P* < 0.05) (One-way ANOVA).

To understand the biological significance of Ca^2+^/CaM interaction with GTL1 on the regulation of *SDD1* promoter-binding activity, we hypothesized that Ca^2+^/CaM-dependent inhibition of the GTL1/*SDD1* promoter complex interaction is a key signal transduction mechanism to activate (derepress) *SDD1* expression in response to water-deficit stress. Indeed, transcript abundance of *SDD1* mRNA is up-regulated in response to water-deficit stress when *DREB2A*, a drought-induced marker gene, was also up-regulated (Fig. 5a). Next, to determine if hyperosmotic stress regulates *SDD1* transcription through GTL1 and Ca^2+^ and CaM signaling, we first established the 2 kb *SDD1* promoter-reporter system (Fig. 5b). SDD1-LUC activity was higher in *gtl1-4* than in wild-type (Col-0) protoplasts, whereas *GTL1* promoter-driven *GTL1-GFP* expression in *gtl1-4* (*GTL1*/*gtl1-4*) protoplasts repressed *SDD1* expression to a level comparable to wild-type protoplasts (Fig. 5b), which is consistent with previous results in mature leaves^26,27^. The *SDD1* promoter-reporter system established that the 2 kb promoter fragment of *SDD1* including the GT3 box is sufficient for *SDD1* expression and GTL1 transrepression.

We next determined the involvement of hyperosmotic stress-induced Ca^2+^ and CaM signaling in the transcriptional regulation of *SDD1*. *SDD1* expression in wild type protoplasts was induced by hyperosmotic stress (200 mM mannitol addition to the protoplast incubation solution), which was partially inhibited by pretreatment with either the Ca^2+^ channel blocker gadolinium ion (Gd^3+^) or the CaM antagonist W7 and abrogated when incubated with both (Fig. 5c). This result indicates that hyperosmotic stress-induced *SDD1* expression is Ca^2+^- and CaM-dependent. Constitutive *SDD1* expression was evident in *gtl1-4* protoplasts without hyperosmotic treatment (Fig. 5c), confirming that GTL1 transrepresses *SDD1* expression^27^. Hyperosmotic stress resulted in a slight increase in *SDD1* expression in *gtl1-4*. W7, Gd^3+^, or Gd^3+^ + W7 pretreatment only marginally reduced *SDD1* expression in *gtl1-4* plants compared to the response in Col-0 (Fig. 5c). This could be due to involvement of a GTL1-independent pathway or other members of the GT-2 family in the regulation of *SDD1* expression. A defect of Ca^2+^-dependent hyperosmotic *SDD1* expression in *gtl1-4* was restored in *GTL1-GFP* expressing lines (*gtl1-4*::*GTL1*_*promoter*_:*GTL1*:*GFP*) (Supplementary Fig. S5). These results indicate that GTL1 is required for Ca^2+^- and CaM-dependent hyperosmotic *SDD1* expression. Together, these results indicate that hyperosmotic stress induces *SDD1* expression through Ca^2+^/CaM signaling and by attenuating GTL1 transrepressor activity.

To determine the *in vivo* direct outcome of the Ca^2+^/CaM-binding function on GTL1 DNA-binding activity, *SDD1* promoter*-LUC* reporter activity in response to hyperosmotic stress was compared by expressing wild-type GTL1 or GTL1[H94E]. Hyperosmolality increased *SDD1* expression in wild-type and *GTL1*-expressing *gtl1-4* protoplasts but not in *GTL1*[*H94E*]-expressing *gtl1-4* protoplasts (Fig. 5d). Moreover, *GTL1*[*H94E*] expression suppressed constitutive *SDD1* expression in *gtl1-4* protoplasts (Fig. 5d). Together, these results indicate that *SDD1* expression is activated or derepressed in response to hyperosmotic stress via allosteric inhibition of GTL1 docking to the *SDD1* promoter by Ca^2+^/CaM.

### Water-deficit stress represses stomatal development in a Ca^2+^/CaM- and GTL1-dependent manner

**Figure 6:**
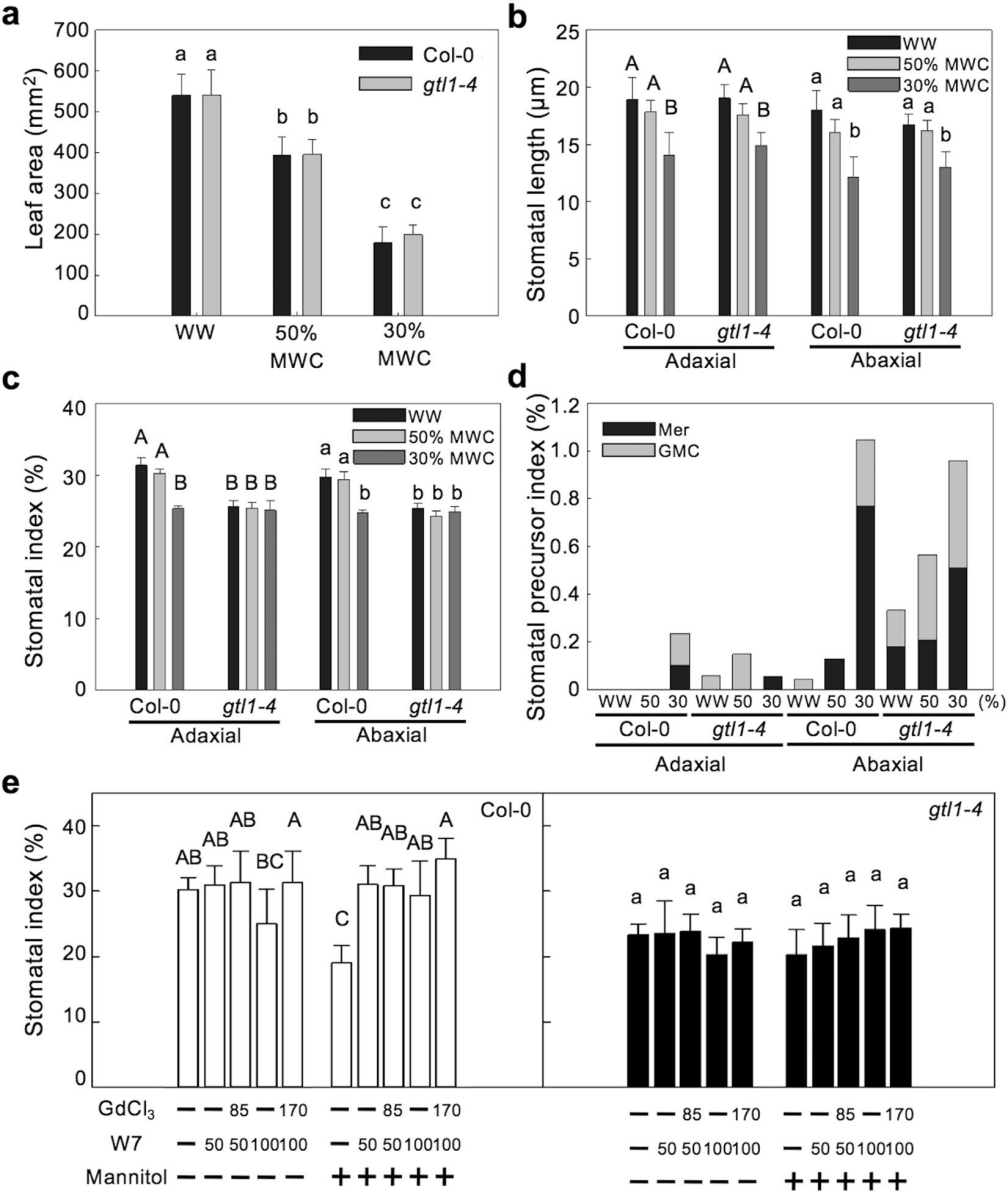
Stomatal development is repressed under severe water-deficit conditions through Ca^2+^/CaM and the GTL1 transrepressor. (**a**) Leaf area of Col-0 and *gtl1-4* plants grown under well-watered (WW), mild water-deficit (50% media water content, MWC), and severe water-deficit (30% MWC) conditions. These leaves were used to quantify stomatal length (**b**), stomatal index (**c**), and stomatal precursor index (**d**) in adaxial and abaxial leaf surfaces of Col-0 and *gtl1-4* plants. Adaxial (upper case) and abaxial (lower case) leaves were analyzed separately for the statistical comparisons. (**e**) Stomatal index of the abaxial surface was quantified in Col-0 and *gtl1-4* plants grown under 0 and 200 mM mannitol conditions with or without W7 (50 and 100 μM) and GdCl_3_ (85 and 170 μM). Col-0 (left panel) and *gtl1-4* (right panel) were analyzed separately for the statistical comparisons. Data shown are the means with SD for 8–12 replicates (**a**–**d**) and 7 replicates (**e**). Columns with the same letters above are not significantly different from each other based on Tukey’s Honestly Significant Difference (HSD) test (*P* < 0.05) (Two-way ANOVA).

Hyperosmotic stress inhibits stomatal development in *Arabidopsis*^6,39^. Overexpression of *SDD1* represses stomatal development in species including *Arabidopsis*, tomato, and maize^40–42^. We have shown that hyperosmotic stress results in up-regulated *SDD1* expression in a Ca^2+^/CaM- and GTL1-dependent manner (Fig. 5). This suggests that water-deficit stress may repress stomatal development through Ca^2+^/CaM-dependent inhibition of the GTL1 transrepressor in plants. To test whether osmotic stress-induced repression of stomatal development is dependent on GTL1, we quantified changes in stomatal development in wild type and *gtl1-4* plants in response to water-deficit stress (Supplementary Fig. S6). The presence of a significant water stress was confirmed by reductions in leaf area (Fig. 6a) and stomatal length (Fig. 6b), both of which are well-established indicators of water stress^39^.

Stomatal index was reduced in *gtl1-4* plants compared to wild type plants (Fig. 6c) as previously reported^27^. We also observed a reduction in stomatal development in severely water-stressed Col-0 plants, evidenced by a decrease of stomatal index in both adaxial and abaxial leaves (Fig. 6c). Mild water-deficit stress (50% MWC) resulted in reduced leaf area, but no change in stomatal index, suggesting that inhibition of stomatal development depends on the severity of the stress. In addition, the number of stomatal precursor cells produced by asymmetric divisions (meristemoids and guard mother cells) was increased in response to severe water-deficit stress (Fig. 6d). In contrast to the water-deficit-induced reduction in stomatal index in Col-0 plants, stomatal development in *gtl1-4* was insensitive to severe water-deficit stress (Fig. 6c), indicating that GTL1 is required for water-deficit stress-induced repression of stomatal development.

To further test the hypothesis that water-deficit stress-repressed stomatal development is dependent on Ca^2+^/CaM signaling, plants were grown on media containing the CaM antagonist W7 alone or with GdCl_3_ with or without mannitol to induce osmotic stress. A lower concentration of W7 (50 and 100 µM), relative to protoplast assays, was used to determine the effects on stomatal development in seedlings without major effects on growth. W7, whether alone or in combination with GdCl_3_, did not significantly affect stomatal index of Col-0 under water-sufficient conditions. However, W7 completely rescued the water-deficit-induced repression of stomatal development regardless of GdCl_3_ treatment (Fig. 6e). There was no effect of W7 or mannitol on stomatal index of *gtl1-4* plants (Fig. 6e). Leaf area was lower in both Col-0 and *gtl1-4* when grown with mannitol, and this phenotype was not rescued by W7 (Supplementary Fig. S7). Together, these results indicate that water-deficit stress results in decreased stomatal development through Ca^2+^/CaM signaling, and that GTL1 is required for transducing the osmotic stress signal to the repression of stomatal development.

## Discussion

**Figure 7:**
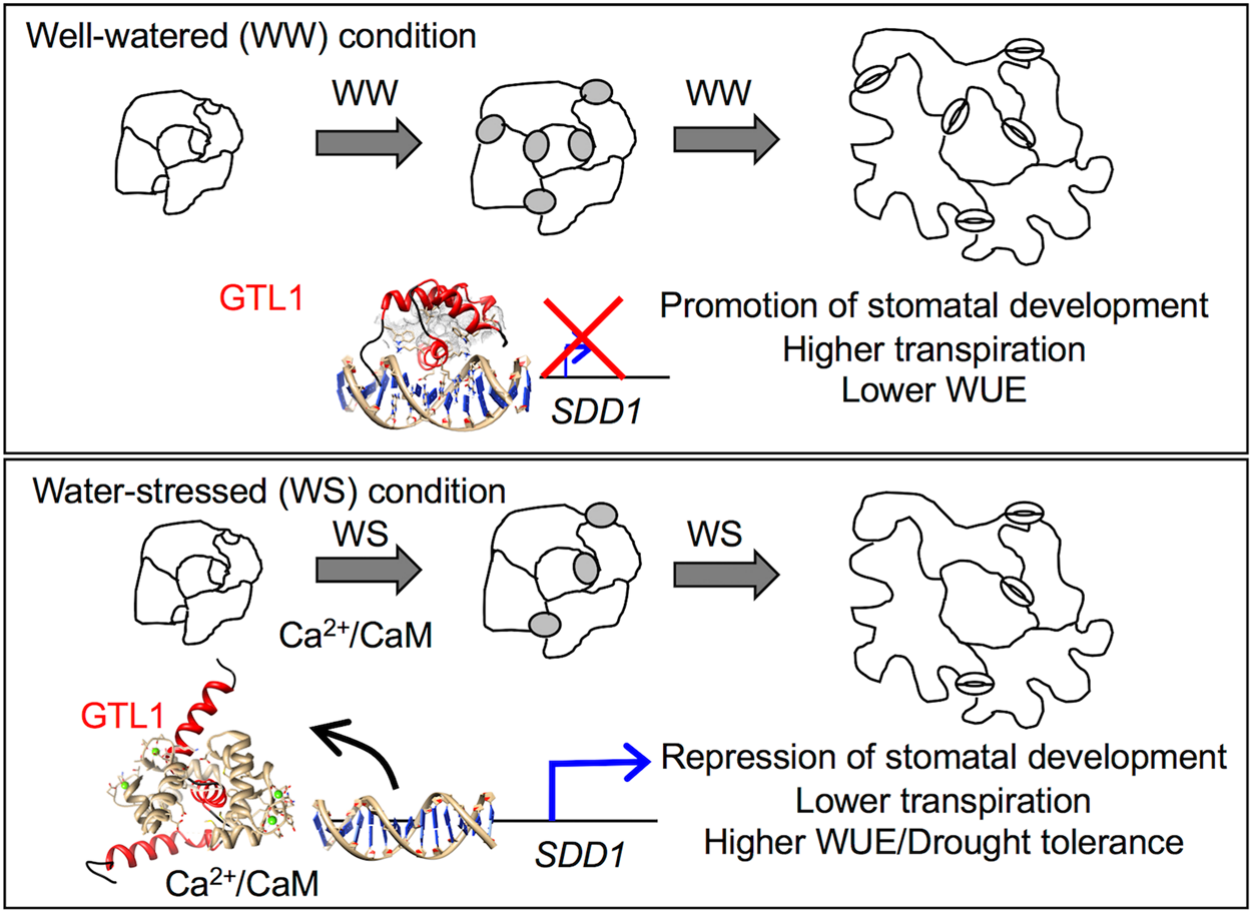
A Ca^2+^/CaM-regulated GTL1 transrepressor is a transcriptional switch to control stomatal development, transpiration, and water-use efficiency in plants. Proposed model for Ca^2+^/CaM-regulated transcriptional switch to repress stomatal development and to improve water-use efficiency and drought tolerance through GTL1.

Hyperosmotic stress-induced Ca^2+^/CaM signaling requires a rapid and efficient signal transduction mechanism to modulate global transcriptional regulation to cope with water deficit during drought stress. The widely accepted model of this signal transduction mechanism is that Ca^2+^/CaM directly interacts with transcription factors to modulate their activity and regulate transcription. However, molecular and structural mechanisms of this modulation specifically on a transcriptional repressor are largely unknown. In this set of experiments, we have demonstrated a mechanism of Ca^2+^/CaM-dependent allosteric control of the GTL1 transcriptional repressor that directly controls its DNA-binding activity in response to hyperosmotic stress (Fig. 7). Under water-sufficient conditions, GTL1 binds to the *SDD1* promoter and represses *SDD1* expression, resulting in maintained stomatal development. Water-deficit induces a transient increase of intracellular Ca^2+^ level^36,38,43,44^, which is transduced by CaM that directly binds to GTL1, destabilizes the trihelical structure of the DNA-binding domain, and facilitates dissociation of GTL1 from the *SDD1* promoter. This novel signal transduction mechanism leads to transcriptional derepression (activation) of *SDD1*, a negative regulator of stomatal development, and thus inhibits stomatal development to reduce transpirational water loss under water-deficit conditions as an acclimation response (Fig. 7).

A prototypical CaM-binding domain exists in the basic amphipathic region of GTL1N α2 (Fig. 1b and Supplementary Fig. S8). It is well known that Ca^2+^/CaM binds to target peptides via hydrophobic and electrostatic interactions^9,34,45^. Ca^2+^ binding to CaM causes a conformational change from a dumbbell shape to a globular shape, which exposes hydrophobic pockets and negatively charged conserved glutamate residues that can interact with protein target regions such as the basic amphipathic α2 helix of GTL1N (Supplementary Fig. S8). The prediction of the GTL1N α2-Ca^2+^/CaM complex suggests that α2 basic residues interact with conserved acidic glutamate residues of CaM through electrostatic interactions (Supplementary Fig. S8)^9,11,45^. The α2 hydrophobic residues interact with the hydrophobic pocket of Ca^2+^/CaM. Since α2 hydrophobic residues are also important for the formation of the hydrophobic core and GTL1N DNA-binding activity (Fig. 3), Ca^2+^/CaM binding to the α2 may interfere with the formation of the hydrophobic core and prevent binding to the *SDD1* promoter. Our results demonstrate that Ca^2+^/CaM binds to the α2 inner-core hydrophobic residues, which destabilizes GTL1N tertiary structure by conformational change, preventing *SDD1* promoter docking, which results in transcriptional derepression of *SDD1* expression.

This allostery is in contrast to the Ca^2+^/CaM steric hindrance mechanism of E proteins in which Ca^2+^/CaM blocks promoter binding of E proteins but does not dissociate the E protein-promoter complex in animals^12^. Because GTL1 is associated with the *SDD1* promoter, resulting in repression under well-watered conditions, this allosteric mechanism allows for dissociation of GTL1 from the promoter and the prevention of free GTL1 binding to the promoter, thereby maintaining *SDD1* activation through both mechanisms. Allostery can involve large or subtle conformational change or can function without conformational change by redistributing electrostatic interactions necessary for binding interface^46^. Whether Ca^2+^/CaM-mediated allosteric regulation of GTL1 DNA-binding activity requires conformational change of trihelical tertiary structure is unknown without analyzing the solution structure of the Ca^2+^/CaM-GTL1 complex. It is, however, well-accepted that allostery is one of the fundamental molecular mechanisms in cellular signaling pathways of all living organisms^47^. We posit that the allosteric mechanism may fine-tune transcriptional regulation that is necessary for precise gene expression required when an organism must decode different signatures encoded by environmental changes necessary for adaptive fitness^48^.

Water deficit in soil results in decreased cell turgor pressure and imposes osmotic stress on plant cells, which activates various acclimation responses, including physiological and developmental changes^49^. Mannitol-induced hyperosmotic stress has been reported to induce cytosolic and nuclear Ca^2+^ transients in tobacco and *Arabidopsis*^36,38,43,44^. Hyperosmotic stress also results in decreased stomatal development in *Arabidopsis*^6,39^. Consistent with these reports, our study also showed that water-deficit stress repressed stomatal development as evidenced by a reduction of stomatal index (Fig. 7). However, in *gtl1-4* plants, stomatal index was not further decreased by mannitol, indicating that GTL1 is required for transducing the signal from the osmotic stress to the modulation of stomatal development. We showed that the Ca^2+^/CaM-GTL1-*SDD1* modular relay system allows for transduction of water-deficit (e.g. turgor reduction or low water potential) sensing. When water is sufficient, GTL1 transrepression of *SDD1* (a negative regulator of guard cell lineage) is necessary to facilitate stomatal development^27,28^. The Ca^2+^/CaM-GTL1-*SDD1* module appears to be a transcriptional switch mechanism through which plants can acclimate to reduced water availability via a repression of stomatal development (Fig. 7). Since YODA (a MAPKKK) is known to act downstream of SDD1, we posit that the Ca^2+^/CaM-regulated transcriptional switch through the GTL1-*SDD1* module is the most upstream signal transduction pathway responsible for this response identified to date. Whether the function of SDD1 is to send a signal in response to water deficit stress remains to be tested.

A Ca^2+^-dependent transcriptional derepression mechanism can be considered to be an efficient signal transduction mechanism for rapid activation of acclimation genes^17,19^. Recently, a similar signal transduction mechanism through the SR1/CAMTA3 transrepressor has been reported in *Arabidopsis*. SR1/CAMTA3 is a Ca^2+^/CaM-binding transcriptional repressor that suppresses the expression of salicylic acid (SA)-related genes involved in plant immunity^14^. Ca^2+^/CaM binding to AtSR1/CAMTA3 is required for repression of the immune response. Low temperature induces expression of SA-related genes through de-repression of SR1/CAMTA3 activity by Ca^2+^/CaM^17,20^. However, the CaM-binding domain is located in the C-terminus, while the DNA-binding domain is located in the N-terminus. It is not known how Ca^2+^/CaM activates CAMTA transcriptional activity or DNA-binding activity. This report together with our results suggest that Ca^2+^/CaM-regulated transcriptional derepression is a common signal transduction mechanism in response to various environmental stresses.

The capacity for plants to sense water-deficit through the Ca^2+^/CaM-GTL1-*SDD1* module and to transduce this signal to a developmental program may be an important mechanism to modulate plant water use. The presence of GTL1 orthologs in a large number of diverse species suggests that this acclimation mechanism is conserved among higher plants^27,28^. Supporting this notion, TaGT2L1D, a GTL1 ortholog in wheat, is also a transrepressor of *SDD1* to negatively regulate stomatal development^30^. We reported that the poplar GTL1 ortholog interacts with Ca^2+^/CaM through the C-terminal DNA-binding domain that binds to the GT2 box of the poplar *SDD1* promoter^26^. It is interesting that the Ca^2+^/CaM-binding domain of AtGT2L is located in the α2 helix of the C-terminal trihelical domain, whereas in PtaGTL1 it is located in the α1 of the C-terminal trihelical domain. Some of the GT2 family do not have Ca^2+^/CaM-binding domains within their trihelical domain due to a change of residue to an acidic residue (Fig. 2b). This suggests that the Ca^2+^/CaM-binding property might have evolved in specific members of the GT2 family, such as GTL1. The Ca^2+^/CaM-binding property of the trihelical DNA-binding domain in GTL1 may have evolved to activate *SDD1* expression by disrupting DNA-binding activity under selective pressure as a drought adaptation mechanism. Future work will reveal the evolutionary/adaptive significance of the allosteric inhibition mechanism in GTL1 orthologs to improve crop production under limited water availability.

## Methods

### Plant materials and growth conditions

The *Arabidopsis thaliana* genetic resources used in this study were wild type Columbia-0 (Col-0), *gtl1-4* (SALK_005972), and transgenic plants expressing *GTL1*_*promoter*_*:GTL1:GFP* in *gtl1-4*^27^. Plants were grown on an MS-based medium (1xMS, 2% sucrose, 2.5 mM MES, pH 5.7, 0.5% agar) under fluorescent lights with a light level of ca. 60 µmol quanta m^−2^ s^−1^ for 4 weeks (temperature: 22 °C [light]/18 °C [dark]; photoperiod: 16 h [light]/8 h [dark]). For stomatal development analysis in soil-grown plants, Col-0 and *gtl1-4* seeds were germinated in a 1:2 soil mix of Turface (PROFILE Products LLC) and Fafard F2 soilless media (Sungro Horticulture) in SC7 Ray Leach ‘Cone-Tainers’ (Stuewe & Sons Inc.). Plants were grown in the greenhouse under an 8 hour photoperiod, with light intensity of 150 µmol m^−2^ s^−1^, day/night temperature of 23/18 °C, and relative humidity of 65%. For stomatal development analysis in plate-grown plants, Col-0 and *gtl1-4* seeds were plated (8–12 plants per genotype per plate) in quadrants on agar plates containing one or more of 200 mM mannitol, 50 or 100 µM W7, and 85 or 170 µM GdCl_3_. Seedlings were grown under a 16 hour photoperiod at 150 µmol m^−2^ s^−1^ for 6 weeks.

### Plasmid construction

*SDD1*_*promoter*_*:LUC* was constructed in the 5′GAL4:LUC vector by replacing the GAL4 binding site with the 2 kb *SDD1* promoter^50^. The 2 kb *SDD1* promoter including the GT3 box and the 5′ untranslated region was amplified with designated primers (SDD1p-F-EcoRV and SDD1p-R-NcoI) and was fused to the firefly luciferase gene (*LUC*) and nopaline synthase (*NOS*) termination signal (*NOS-t*) to produce an *SDD1* reporter (*SDD1-LUC*) for protoplast transactivation assays. The promoter containing 5′GAL4 from 5′GAL4:LUC vector was removed by Hind III (with blunt-end generation by Klenow) and NcoI. The amplified *SDD1* promoter digested by EcoRV (blunt-end) and NcoI was inserted to the HindIII (blunt-end)/NcoI site.

Two *GTL1* coding fragments (GTL1N and GTL1Δdel) were amplified by designated primers (GTL1N, N-F-EcoRI and N-R-PstI; GTL1Δdel, N-F-EcoRI and del-R-PstI). Two site-specific mutations (V95D and H94E) were amplified by designated primers (V95D-F, V95D-R, H94E-F, and H94E-R with N-F-EcoRI and N-R-PstI) by PCR-based site-directed mutagenesis. The amplified fragments and mutations were inserted into the EcoRI and PstI sites in the pMAL-C2 vector to produce MBP-fusion proteins. For the transient protoplast assay, *HA:GTL1* and *HA:GTL1*[*H94E*], driven by the 35S promoter, were constructed in the p326:HAN vector^51^. *GTL1* or *GTL1*[*H94E*] coding fragment was amplified by designated primers (GTL1, GTL1-F-XmaI and GTL1-R-EcoRI; GTL1[H94E], GTL1-F-XmaI and H94E-R, and H94E-F and GTL1-R-EcoRI). p326:HAN vector was digested with KpnI (with blunt-end generation by Klenow) and XmaI. Amplified *GTL1* and *GTL1*[*H94E*] products were digested with EcoRI (with blunt-end generation by Klenow) and XmaI, which was inserted into KpnI (blunt end)/XmaI sites.

*Arabidopsis* CaM2 was amplified by designated primers (AtCaM2-F and AtCaM2-R). The amplified fragments were inserted into the BamHI and XhoI sites of pCMX-PL2-NterHA using HiFi Assembly (NEB). Sequence information for all designated primers used for plasmid construction is provided in Supplementary Table S1.

### *Arabidopsis* protoplast transient expression and luciferase reporter assay

Protoplasts were isolated from 4-week-old plants as described previously^52^ with modifications. Shoots from 150 seedlings were incubated in 30 mL of 1 M mannitol solution for 30 min and then incubated in 20 mL of enzyme solution (1% cellulose R-10, 0.25% macerozyme R-10, 500 mM mannitol, 1 mM CaCl_2_, 10 mM MES, 20 mg BSA) for 12 h to digest cell walls. The protoplast mixture was filtered through 100 mm mesh and transferred onto 20 mL of 21% sucrose solution, which was centrifuged at 57 *g* for 10 min in a swinging bucket rotor. Approximately 10 mL of protoplasts at the interface were transferred to 30 mL of W5 protoplast stabilization solution (154 mM NaCl, 125 mM CaCl_2_, 5 mM KCl, 2 mM MES, pH 5.7) and centrifuged at 43 *g* for 6 min. Supernatant was removed, replaced by 30 mL of W5 solution, and incubated on ice for 6 h to stabilize protoplast.

*SDD1*_*promoter*_*:LUC* (29 mg), *35S*_*promoter*_*:LUC* (1 mg), *HA:GTL1* (5 mg), or *HA:GTL1*[*H94E*] (5 mg) was transformed into protoplasts using the polyethylene glycol (PEG)-mediated transformation method^52^. First, W5 solution was replaced by mannitol solution (400 mM mannitol, 30 mM MgCl_2_, and 5 mM MES, pH 5.6). Plasmids (30 ml) were added to 300 ml of protoplast MaMg solution. Then, 300 ml of PEG solution (400 mM mannitol, 100 mM Ca(NO_3_)_2_, 40% PEG6000) was added, mixed by inverting for 1.5 min, and incubated at room temperature for 30 min. PEG protoplast solution was slowly diluted with 5 ml of W5 solution by adding 1 ml of solution every 2 min. The protoplast solution was centrifuged at 43 *g* for 4 min. Supernatant was completely removed, replaced by 2 ml of W5 solution, and incubated at room temperature for 12 h. To induce hyperosmotic stress, 200 mM mannitol was added and protoplasts were incubated for 1 h. To inhibit Ca^2+^ or CaM signaling, protoplasts were pre-incubated in 1 mM GdCl_3_, 0.5 mM LaCl_3_, or 600 mM W7 (*N*-(6-aminohexyl)-5-chloro-1-naphthelenesulfon-amide-hydrochloride) for 10 min prior to mannitol treatment. After incubation with mannitol, protoplasts were harvested by centrifugation at 57 *g* for 2 min, then frozen in liquid nitrogen. Frozen protoplasts were lysed with passive lysis buffer (Promega) and the SDD1-LUC and RrLUC activities were measured using a dual-luciferase assay kit according to the manufacturer’s instructions (Promega) using a luminometer (TD20/20). Relative SDD1-LUC activity was shown as 100× (SDD1-LUC activity/RrLUC activity). Osmotic potential (*ψ*_*s*_) of the osmotic stabilization solution (W5; (*ψ*_*s*_: −1.19 MPa) and hyperosmotic solution (W5 with 200 mM mannitol; *ψ*_*s*_: −1.73 MPa) was calculated by the equation (*ψ*_*s*_ = −CRT; C = osmolality, R = gas constant, T = Kelvin temperature). Osmolality of the solution was measured using a vapor pressure osmometer (Wescor 5200).

### Purification of recombinant proteins

MBP and MBP-fusion proteins (GTL1N, GTL1C, GTL1Δdel, GTL1N[L91R], GTL1N[W92R], GTL1N[H94E], and GTL1N[V95D]) were purified by amylose resin (NEB) as described previously^27^.

### CaM-binding assays

CaM-binding assays were performed using bacteria lysates expressing MBP-fusion proteins and *in vitro-*translated HA-AtCaM2 as described previously^53^ with the following the modifications. Briefly, MBP fusion proteins were expressed in *Escherichia coli* strain Rosetta (DE3) carrying pMAL-c2 vectors. After harvesting by centrifugation, the cells were lysed by French press in lysis buffer containing 50 mM Tris-HCl, pH 7.5, 100 mM NaCl, 1% DMSO, 2 mM DTT, and protease inhibitor cocktail (Sigma-Aldrich). The cell extract was prepared by centrifugation at 10,000 g for 20 min at 4 °C and cleared by filtration. The clear lysates were incubated with amylose resin at 4 °C for 2 h. The beads with immobilized MBP fusion proteins were washed with wash buffer containing 50 mM Tris-HCl, pH 7.5, 100 mM NaCl, 1% DMSO, 2 mM DTT, and 0.1% Nonidet P-40. AtCaM2 with an N-terminal HA tag was synthesized using plasmid pCMX-PL2 and the TNT T7 Coupled Reticulocyte Lysate System (Promega) according to the manufacturer’s protocol. The *in vitro-*translated HA-AtCaM2 was diluted with CaM-binding buffer (50 mM Tris-HCl, pH 7.5, 100 mM NaCl, 1% DMSO, 2 mM DTT, and 2 mM CaCl_2_) and incubated with the affinity-purified MBP fusion proteins immobilized on the beads at 4 °C for 2 h. The beads were then washed four times with wash buffer. Bound HA-AtCaM2 was eluted by boiling in 1x SDS sample buffer and subjected to 12% SDS-PAGE. Input and immunoprecipitated HA-AtCaM2 proteins were detected by immunoblots using rabbit polyclonal anti-HA antibodies (Abcam) and the Enhanced Chemiluminescence Plus protein gel blotting detection system (GE Healthcare). The amount of MBP fusion proteins used in each CaM-binding assay was separated by another 8% SDS-PAGE gel and visualized by staining with Coomassie Brilliant Blue.

CaM-overlay assays using HRP-conjugated AtCaM2 proteins were performed as described previously^54^. Briefly, MBP fusion GTL1 proteins were separated by SDS-PAGE and transferred to two PVDF membranes. After blocking in TBST buffer containing 7% non-fat dry milk, membranes were incubated in the overlay buffer (50 mM imidazole-HCl, pH 7.5 and 150 mM NaCl) containing 1 µg/ml HRP-AtCaM2 in the presence of Ca^2+^ or EGTA for 1 h. The bound HRP-AtCaM2 signal was detected by the Enhanced Chemiluminescence Plus protein gel blotting detection system.

### Electrophoretic mobility shift assay (EMSA)

Purified recombinant proteins were used to determine DNA-binding activity based on interaction with biotin-labeled *Arabidopsis SDD1* promoter fragments that contain the GT3 box. EMSA procedures were as described previously^27^, with the following modifications to determine the effect of Ca^2+^/CaM on DNA-binding activity of recombinant proteins. To determine the effect of CaM on the association between GTL1N and the *SDD1* promoter, human CaM protein (50 or 100 ng) was incubated with recombinant proteins with 2 mM CaCl_2_ or 10 mM EGTA for 1 h. Then, biotin-labeled *SDD1* promoter was added and incubated for 20 min. To determine the CaM effect on the dissociation of GTL1N from the *SDD1* promoter, biotin-labeled *SDD1* promoter was incubated with GTL1N or GTL1N[H94E] for 20 min. Then, CaM protein (100 ng) was added to the GTL1-*SDD1* promoter complex mixture with 2 mM CaCl_2_ and incubated for 1 h. The mixture was separated in a 6% PAGE gel and transferred to a Biodyne B Nylon membrane (Thermo Fisher Scientific). Migration of biotin-labeled *SDD1* promoter was detected by LightShift Chemiluminescent EMSA kit (Thermo Fisher Scientific) according to the manufacturer’s protocol.

### Chromatin immunoprecipitation

ChIP assays were performed using chromatin isolated from protoplasts of 4-week-old *gtl1-4* plants using an EZ ChIP chromatin immunoprecipitation kit (Millipore) as described previously^27^ with the following modifications. Protoplasts were transformed with *HA-GTL1* or *HA-GTL1*[*H94E*] and incubated for 12 h at room temperature. 200 mM mannitol was added to one group (each group includes 3 replicates) to induce hyperosmotic stress for 1 h. Then, formaldehyde was added for crosslinking of chromatin and 0.1 M glycine to quench crosslinking. After centrifuging briefly, protoplasts were resuspended in lysis buffer. Chromatin was sheared to 200~1000 bp fragments by sonication. Isolated and pre-cleared chromatin was incubated with mouse monoclonal anti-HA antibody or mouse IgG for 4 h at 4 °C to immunoprecipitate the chromatin associated with HA-GTL1 or HA-GTL1[H94E]. Immunoprecipitated chromatin was reverse-crosslinked and purified according to the manufacturer’s protocol. Input chromatin (immunoprecipitated by anti-HA antibody or IgG antibody) was used for PCR analysis by the *SDD1* promoter-specific primers (SDD1ChIP-3F and SDD1ChIP-3R)^27^.

### Stomatal development analysis

Following irrigation and drainage of excess water, containers were weighed to obtain media saturated weight (MSW). Plants were irrigated to saturation (well-watered) or water was withheld to 50 or 30% media water content (MWC), calculated as [media fresh weight (MFW) − media dry weight (MDW)]/[media saturated weight (MSW) − MDW] × 100. During the experiment, a MDW of 49 g was used to determine the appropriate amount of water to add to containers, based on prior experiments. After harvesting, containers were dried in a forced-air oven and MDW was obtained for each plant. Final average MWC of the 50 and 30% MWC treatments was Final average MWC of the 50 and 30% MWC treatments was between 38% (prior to watering) and 53% (post-watering), and 25% (prior to watering) and 33% (post-watering), respectively (Supplementary Fig. S6). Once the treatment MWC was reached, containers were wrapped with plastic film to prevent water loss by evaporation. Containers were weighed every two days and re-watered to bring the MWC to target levels (Supplementary Fig. S6). This treatment was maintained for 30 days after the emergence of the target leaf, enabling the leaf to complete development under a given water deficit treatment.

Leaf appearance was tracked with photographs to determine leaf number. Once the target leaves had fully expanded, leaf adaxial and abaxial surfaces were pressed onto cyanoacrylate droplets (Henkel Manufacturing) on glass slides. Impressions were viewed under a BH-2 light microscope (Olympus) at 200× magnification, with a field of view of 0.113 mm^2^. Four images were collected from each surface impression, and cell types were counted in ImageJ. Cell types were distinguished by shape; meristemoids were roughly triangular cells, while long oval cells were identified as guard mother cells (GMCs)^55^. Stomatal index was calculated as the number of stomata/(number of stomata + epidermal cells). A diagonal line was drawn through each image and the 5 stomata closest to this line were used for stomatal length measurements. Length was measured from end to end of the outer cell wall.

To test the necessity of calcium-CaM binding for the inhibition of GTL1-dependent stomatal development under osmotic stress conditions, a pharmacological disruption experiment was performed *in vivo*. Seedlings were grown for 6 weeks, at which point the first true leaf was collected and fixed in a 9:1 ethanol:acetic acid solution. Following clearing, leaf tissue was stored in 85% ethanol. Tissue was rehydrated by transferring to a 70% ethanol solution, then to 70, 50, and 30% methanol solutions, each for ca. one hour. The tissue was then stained with 0.2% toluidine blue and mounted for DIC microscopy in 50% glycerol. All microscopy was done at a 400× magnification. Leaf tissue used for microscopy was also photographed for measurements of leaf area in ImageJ.

### Homology modeling, model validation, and computational docking

Homology modeling of the GTL1 N-terminal trihelix DNA-binding domain (residues 63 to 126) was modeled using the RaptorX web-based server^56^. Structure refinement of the predicted models was performed using ModRefiner^57^. The predicted model of GTL1N was validated by PROCHECK^58^ to check stereochemical quality of a native structure. Ramachandran plot (Supplementary Fig. S9a) and plot statistics (Supplementary Table S2) indicate 95.3% residues in most favored regions, showing a good quality of GTL1N structure. The GTL1N model was further evaluated using the ProSA web server^59^. ProSA calculates the Z-score (−4.13) for the model to show the overall model quality (Supplementary Fig. S9b) and knowledge-based energy plot of residue scores (Supplementary Fig. S9c).

The structure of the GTL1N-*SDD1* promoter complex was determined using the High Ambiguity Driven biomolecular DOCKing (HADDOCK) web server^60^. The active residues of GTL1N involved in docking are chosen based on the NMR structure of GT1 and GT-1 box element and are N61, R62, K109, E113, E116, N117, K120, and K126. The residues around active residues were automatically defined as passive residues. 3D structure of the *SDD1* promoter fragment (5¢-gcttGGTAAAactt-3¢) including a GT3-box was generated by 3D-DART, a DNA structure-modeling server^61^. The GT2 box residues in the *SDD1* promoter fragment (Gua5, Gua6, Thy7, Ade8, Ade9, Ade10) and their complementary residues were considered active residues. A total of 20,000 structures were generated using rigid body energy minimization. The 200 lowest-energy structures were refined by semiflexible refinement and explicit solvent calculations. The model with the best (lowest) HADDOCK score (−175.6 ± 6.2) and root mean square deviation (2.0 ± 1.2) was selected and used for the analysis. All 3D structures were analyzed and visualized by 3D structure viewer UCSF Chimera^62^.

### Statistical analysis

All column graphs were analyzed by Tukey’s honestly significant difference (HSD) test (one-way ANOVA) using SAS 9.2 software (SAS Institute).

## Supplementary information

Supplementary Information
