# Supplementary material for: A Ca2+/CaM-regulated transcriptional switch modulates stomatal development in response to water deficit
Source: Sci Rep. 2019 Aug 22;9:12282. doi: 10.1038/s41598-019-47529-2 (PMC6706580; doi:10.1038/s41598-019-47529-2)
Supplement: Supplementary file 1 — Supplementary Information [file 41598_2019_47529_MOESM1_ESM.pdf]

Supplementary Information

A Ca^{2+}/CaM-regulated transcriptional switch modulates stomatal development in response to water deficit

Chan Yul Yoo[1,†], Noel Mano[2], Aliza Finkler[3], Hua Weng[1], Irene S. Day[4], Anireddy S.N. Reddy[4], B.W. Poovaiah[5], Hillel Fromm[3], Paul M. Hasegawa[1], Michael V. Mickelbart[1,2,*]

[1]Department of Horticulture and Landscape Architecture, Purdue University, West Lafayette, IN 47907, USA; [2]Department of Botany and Plant Pathology, Purdue University, West Lafayette, IN 47907, USA; [3]School of Plant Sciences and Food Security, Faculty of Life Sciences, Tel Aviv University, Tel Aviv, Israel, 6997801; [4]Department of Biology and Program in Cell and Molecular Biology, Colorado State University, Fort Collins, CO 80523, USA; [5]Department of Horticulture, Washington State University, Pullman, WA 99164, USA.

[†]Present address: Department of Botany and Plant Sciences, Institute of Integrative Genome Biology, University of California Riverside, Riverside, CA, 92521, USA

[*]For correspondence (mickelbart@purdue.edu).

Contact of the Corresponding Author:
Michael V. Mickelbart
Department of Botany and Plant Pathology, Purdue University
West Lafayette, IN 47907-2010, USA
Phone: (765) 494-7902
E-mail: mickelbart@purdue.edu

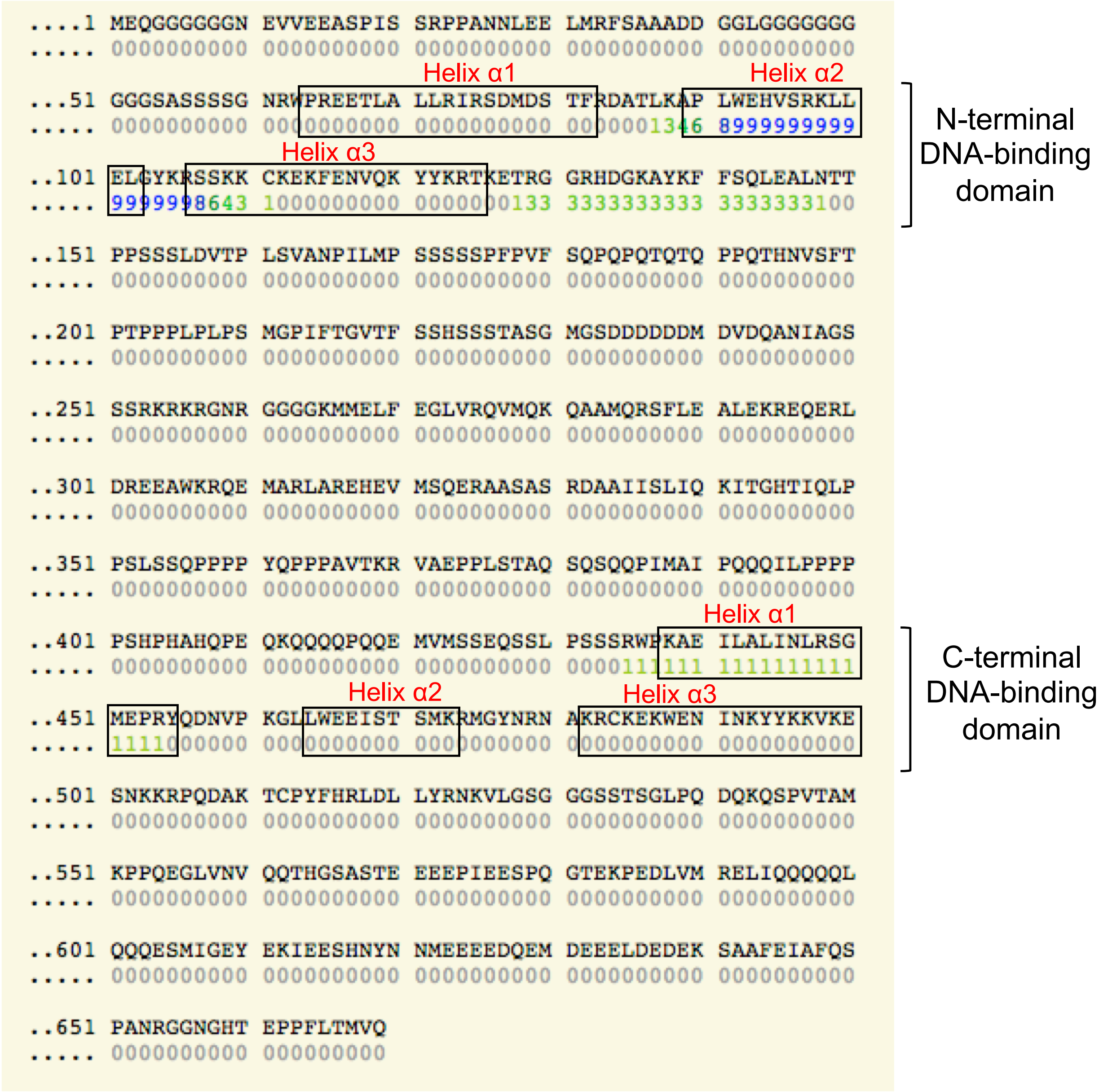

Supplementary Figure S1. Prediction of a calmodulin-binding site in GTL1 suggests a potential binding site in the 2nd helix within the N-terminal DNA-binding domain.

Full-length (669 amino acid residues) GTL1 was analyzed for potential CaM-binding sites in a calmodulin target database (http://calcium.uhnres.utoronto.ca). CaM-binding sites were predicted by multiple characteristics including hydropathy, alpha-helical properties, hydrophobic residue content, and residue charge, which were normalized by scores (0 to 9) and shown below the sequence. A consecutive string of high values indicates a putative calmodulin binding site.

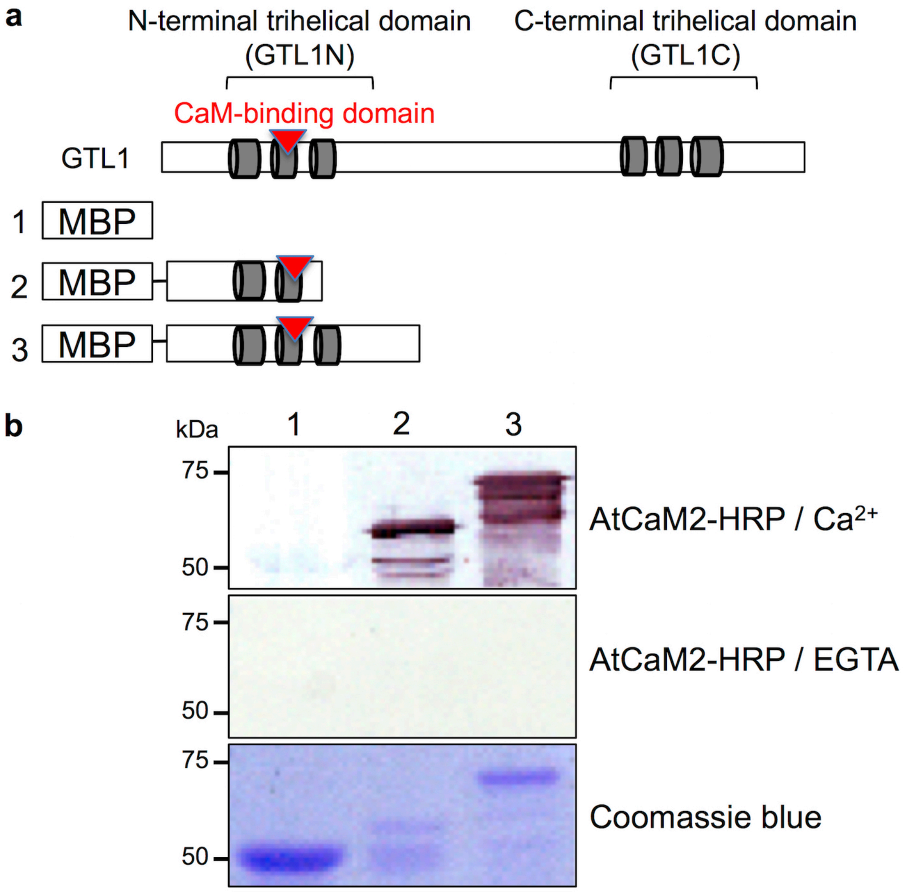

Supplementary Figure S2. Arabidopsis CaM2 directly interacts with the GTL1 N-terminal DNA-binding domain in a Ca^{2+}-dependent manner. (**a**) Schematic illustrations of GTL1 full-length and fragment proteins fused with maltose-binding protein (MBP). Fragment 1 (MBP alone) was used as a negative control. Fragment 2 included the 1st and 2nd helices (CaM-binding domain). Fragment 3 included the 1st, 2nd, and 3rd helices. (**b)** CaM-overlay assay revealed that fragments 2 and 3 interacted with *Arabidopsis* CaM2-HRP (horseradish peroxidase) conjugates only when Ca^{2+} is available.

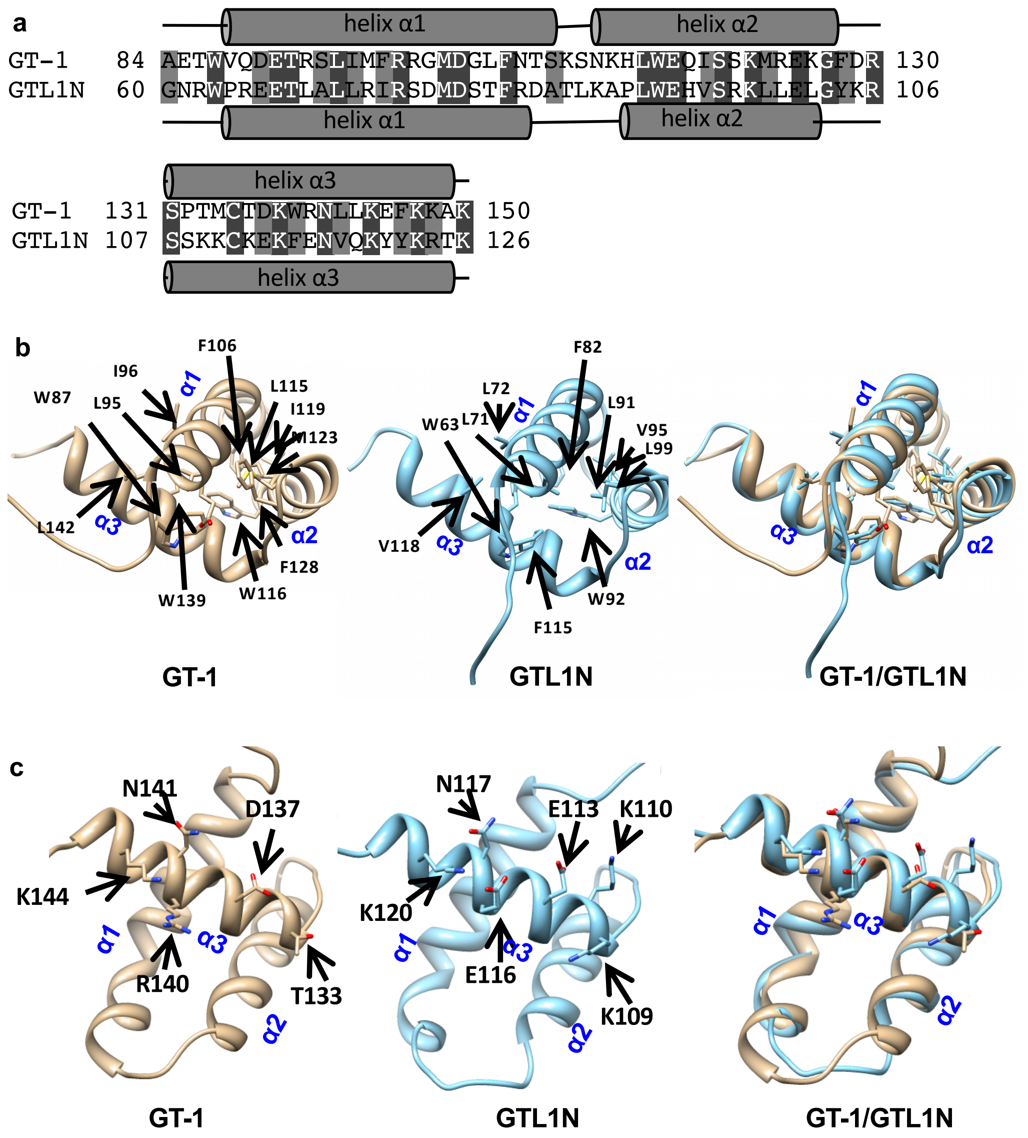

Supplementary Figure S3. GTL1 N-terminal trihelix DNA-binding structure is similar to GT-1 trihelix DNA-binding structure. (**a**) Amino acid alignment of GT-1 and GTL1 trihelix DNA-binding domains. Identical amino acids are highlighted in dark gray and chemically-similar amino acids (e.g. acidic, basic, or non-polar) are highlighted in light gray. The positions for three helices (α1, α2, and α3) are indicated by gray tubes for GT-1 (above) and GTL1 (below). (**b**) Ribbon representations of GT-1 (left), GTL1 (middle), and superposition of the two structures (right). Hydrophobic amino acids forming a hydrophobic core are shown for both GT-1 and GTL1. Superpositions of the two tertiary structures indicates that these hydrophobic residues are located in the identical positions to form a hydrophobic core. (**c**) Ribbon representations of GT-1 (left), GTL1 (middle), and superposition of GT-1/GTL1 (right). Charged amino acids in the 3rd helix important for DNA interaction are shown for both GT-1 and GTL1. Superposition of GT-1/GTL1 indicates that these residues are also located in the same positions to recognize GT-elements in promoter regions.

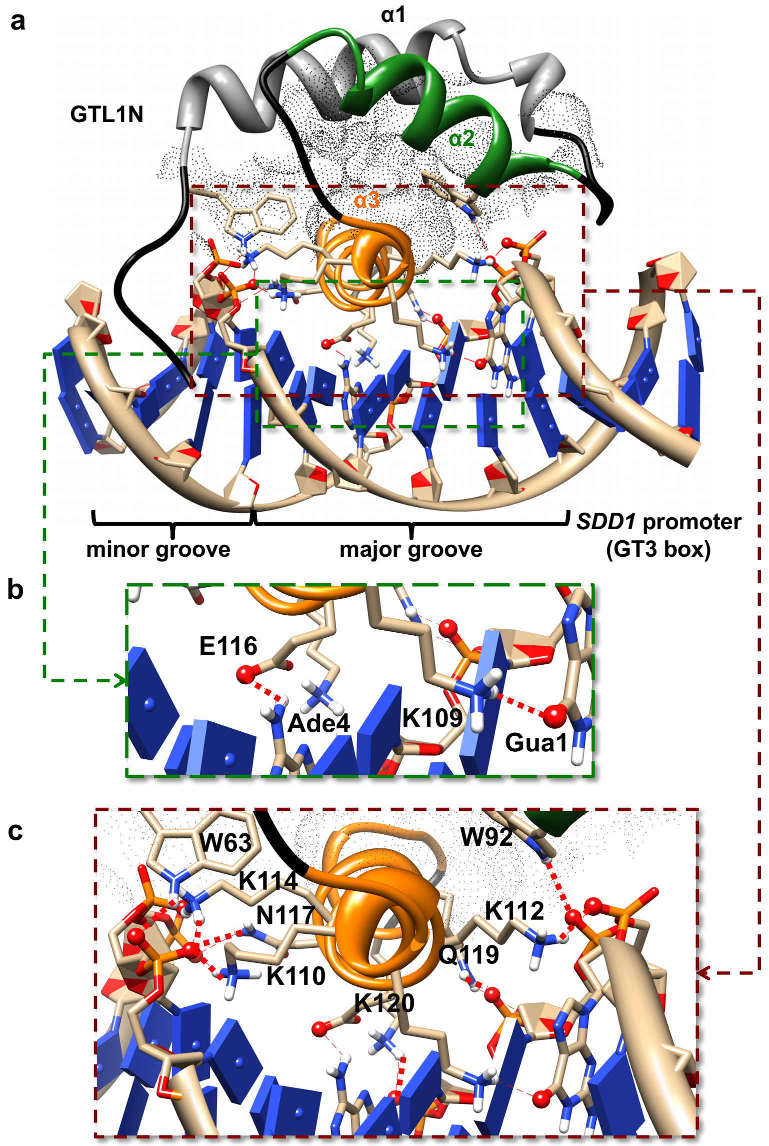

Supplementary Figure S4. GTL1N α3 binds the *SDD1* promoter through GT3 box-specific interactions and the phosphate backbone of DNA. (**a**) A transverse view of the GTL1N-*SDD1* docking structure predicts α3 docking to the major groove of the *SDD1* GT3 box. The HADDOCK computational docking software generated the GTL1N-*SDD1* docking structure. (**b and c**) Amino acid-nucleotide-specific interactions and phosphate backbone contacts involve hydrogen bonds that are indicated by red dashes (H – white, O – red, and N – blue). (**b**) Magnified area displays specific hydrogen bonds between α3 K109 and the 1st Gua nucleotide (2.13 Å distance), and α3 E116 and the 4th Ade nucleotide (3.12 Å). (**c**) The GTL1N-*SDD1* docking structure is stabilized by electrostatic interactions between residues (W63, α2 - W92, and α3 - K110, K112, K114, N117, Q119, and K120) and nucleotide phosphate groups in the major groove.

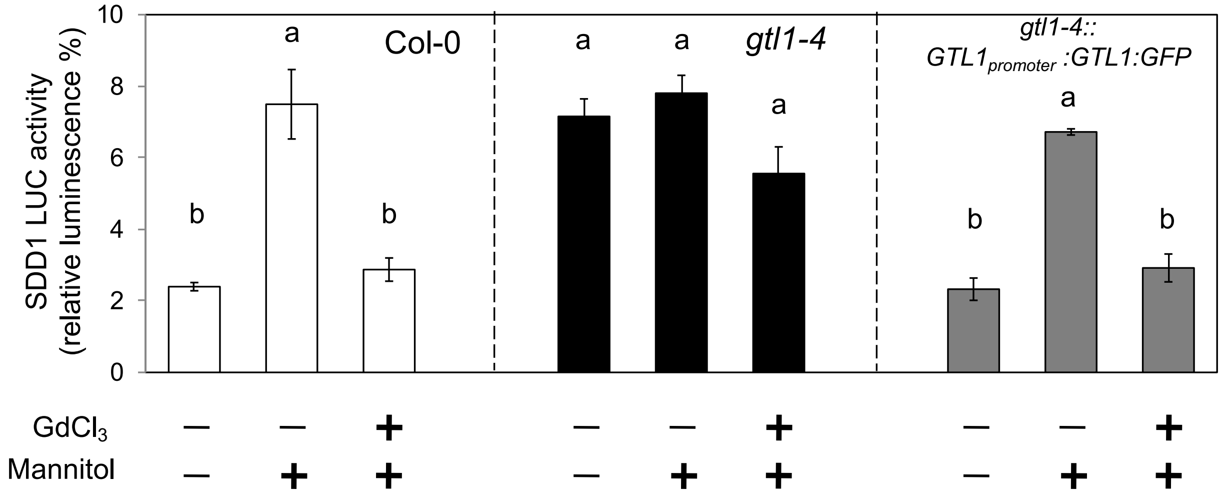

Supplementary Figure S5. GTL1 is required for Ca^{2+}-dependent hyperosmotic stress-induced *SDD1* expression. Relative SDD1-LUC activities were determined in Col-0, *gtl1-4*, and *gtl1-4::GTL1:GFP* protoplasts that were incubated in stabilization solution for 10 min with or without 1 mM $GdCl_3$ prior to 1 h incubation in stabilization or hyperosmotic solution (stabilization solution + 200 mM mannitol). All results shown are mean ± SEM (n = 3). Columns with the same letters above are not significantly different from each other based on Tukey's Honestly Significant Difference (HSD) test ($P < 0.05$) (One-way ANOVA).

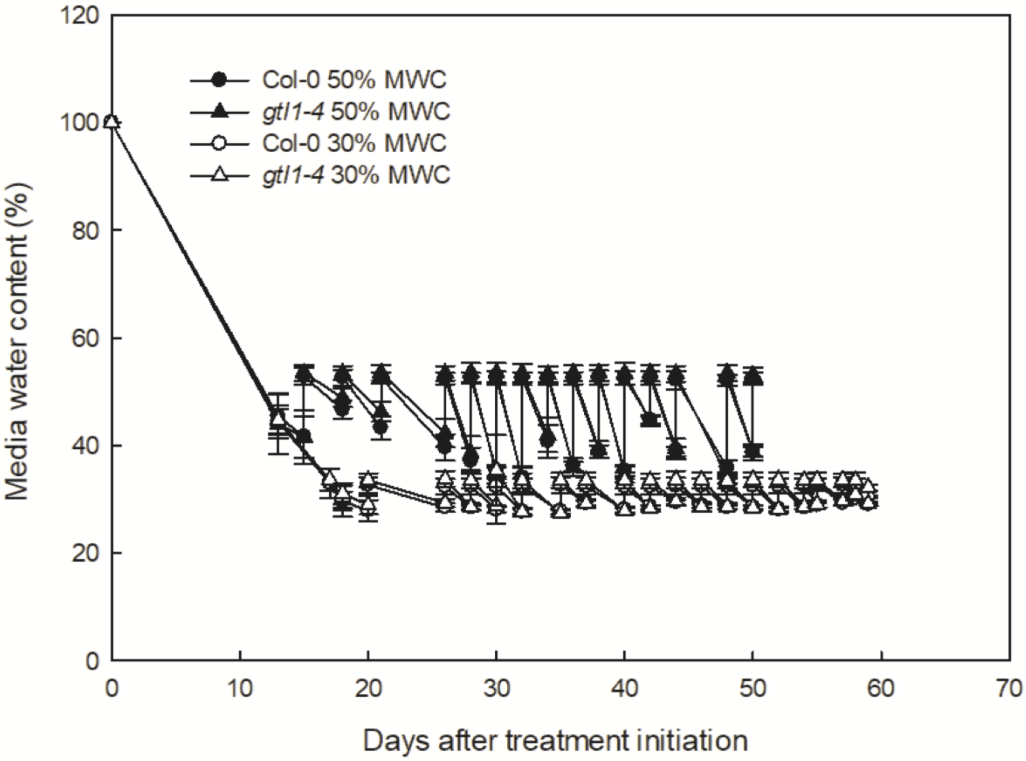

Supplementary Figure S6. Maintenance of media water content (MWC) in Col-0 and *gtl1-4* plants. From the point at which the target MWC (50% or 30%) was reached, MWC was maintained at this level over time by weighing containers and adding water as needed every two days. These treatments were performed for 30 days after the target MWC was reached to enable a leaf to develop from pre-emergence to fully expanded under the target MWC. Well-watered control plants were watered to saturation every 3-6 days as necessary.

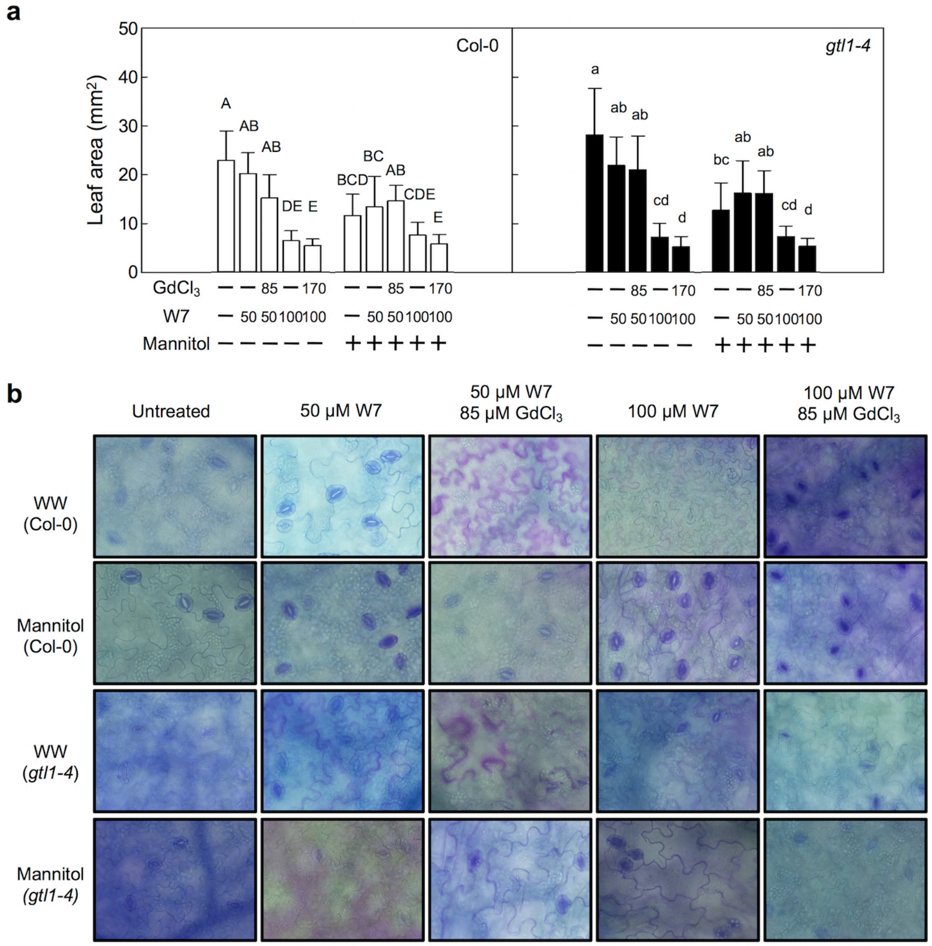

Supplementary Figure S7. Leaf area measurement and representative images of abaxial epidermal layers in Col-0 and *gtl1-4* plants in response to osmotic stress and chemical inhibitor treatment. (**a**) Leaf area was quantified in the first true leaves of Col-0 and *gtl1-4* plants grown under 0 and 200 mM mannitol conditions with or without W7 (50 and 100 μM) and $GdCl_3$ (85 and 170 μM). Col-0 (left panel) and *gtl1-4* (right panel) were analyzed separately for the statistical comparisons. Data shown are the means with SD for 7 replicates. Prior to statistical analysis, data was transformed using a Johnson normalizing function to satisfy the assumption of normality. Columns with the same letters above are not significantly different from each other based on Tukey's Honestly Significant Difference (HSD) test ($P < 0.05$) (Two-way ANOVA). (**b**) Representative abaxial epidermal images of fully expanded first true leaves of Col-0 and *gtl1-4* plants grown under 0 and 200 mM mannitol conditions with or without W7 (50 and 100 μM) and $GdCl_3$ (85 and 170 μM). Leaves were imaged using DIC microscopy.

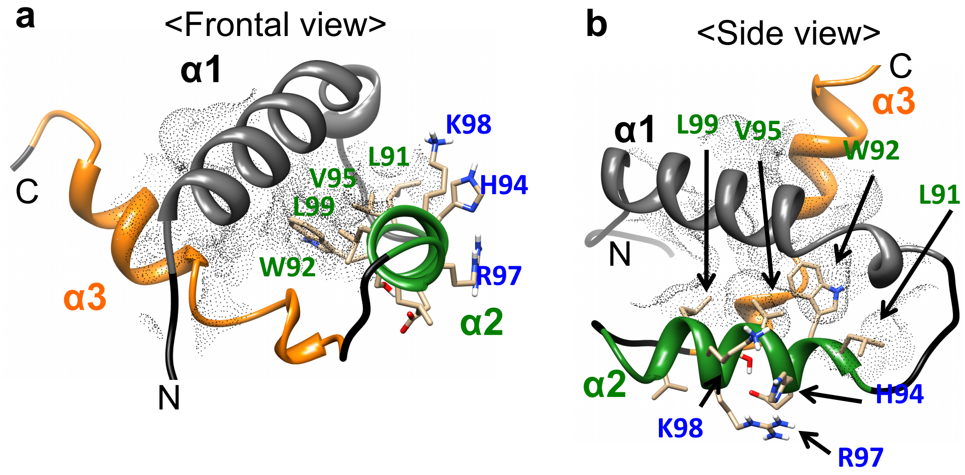

Supplementary Figure S8. Tertiary structure of the GTL1N trihelical domain has a basic, amphipathic α2 helix structure that is predicted to interact with CaM via hydrophobic and electrostatic interactions. (**a and b**) Frontal and side views of GTL1N α2 (green helix) indicates a predicted CaM-binding amphipathic helix with basic (H94, R97, and K98 – blue) and hydrophobic (L91, W92, V95, and L99 – green) residues.

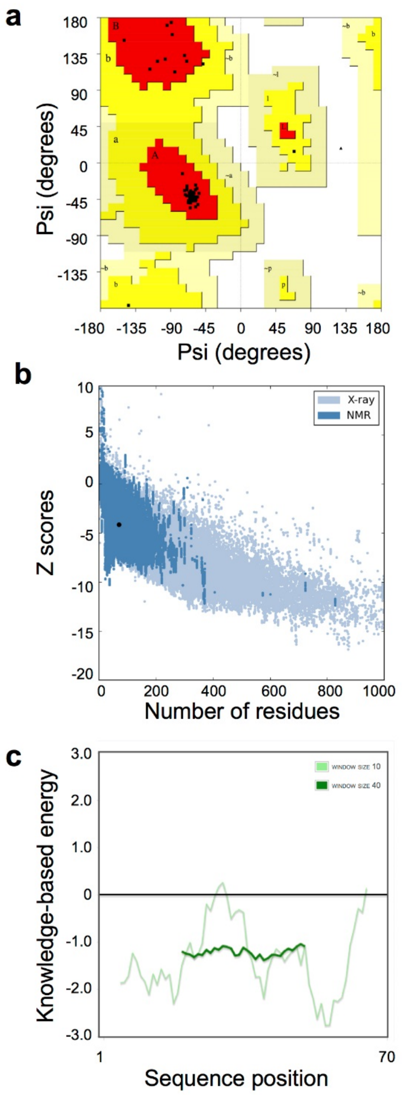

Supplementary Figure S9. Validation of GTL1 N-terminal trihelix DNA-binding three-dimensional structure. (**a**) Ramachandran plot for GTL1N model was generated in PROCHECK. The most favored regions (A, B, L) are colored red. Additional allowed (a, b, l, p), generously allowed (~a, ~b, ~l, ~p), and disallowed regions are indicated as yellow, light yellow, and white fields, respectively. (**b**) ProSA-web Z score of all protein chains in PDB determined by X-ray crystallography (light blue) or NMR-spectroscopy (dark blue). The z-score (-4.13) of GTL1N model is shown (black dot). (**c**) Plot of residue scores of a GTL1N native structure showing average energy of 10 residues (light green) and 40 residues (dark green). Knowledge-based energy plot indicates the local model quality to evaluate model accuracy.

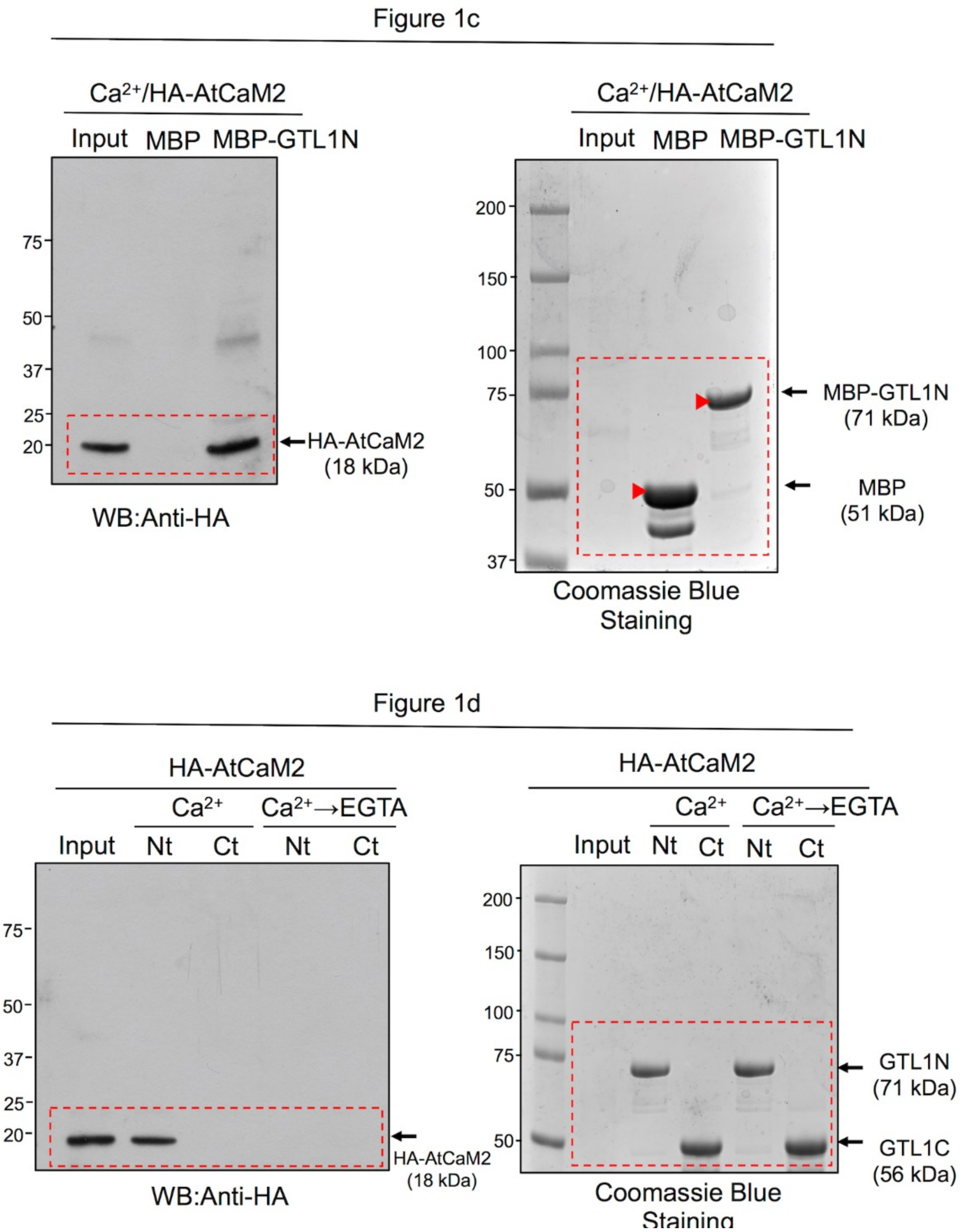

Supplementary Figure S10. Scanned original images of immunoblots and gels.

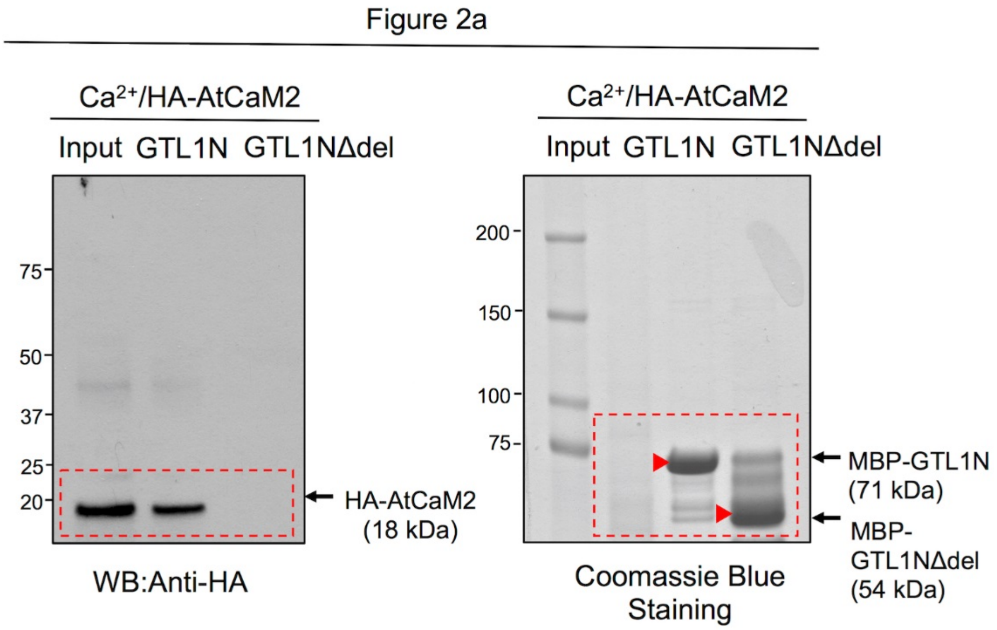

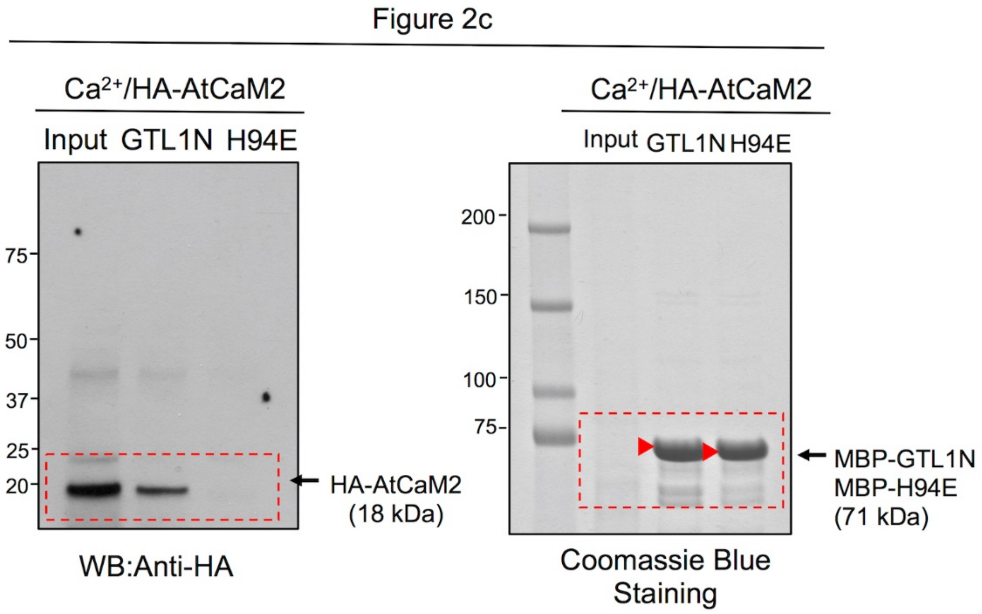

Supplementary Figure S10. (Continued)

Supplementary Table S1. Primer sequence information used for plasmid construction.

SDD1p-F-EcoRV	5'-gatatctgggattgttgggcgaac-3'
SDD1p-R-NcoI	5'-catgccatggtggagagagttaaaaaaaggag-3'
N-F-EcoRI	5'-gaattcatggagcaaggaggaggt-3'
N-R-PstI	5'-ttggctgcagttattccatcattttaccgcctccacc -3'
del-R-PstI	5'-ttggctgcagttatttgagagtagcatcacg-3'
C-F-EcoRI	5'-gaattcatggtcatgagctcggaaca-3'
C-R-PstI	5'-ttggctgcagttactgaaccattgtcaagaaaggt-3'
L91R-F	5'-catgttcccaacgaggagctttgagagtagcatc-3'
L91R-R	5'-caaagctcctcgttgggaacatgtttccaggaag-3'
W92R-F	5'-aaacatgttccctaagaggagctttgagagtag-3'
W92R-R	5'-agctcctcttagggaacatgtttccaggaagctat-3'
H94E-F	5'-tctttgggaagaggtttccaggaagctattgg-3'
H94E-R	5'-tcctggaaacctcttcccaaagaggagctttg-3'
V95D-F	5'-tgggaacatgtttccaggaagctattgga-3'
V95D-R	5'-cttcctggaatcatgttcccaaagaggag-3'
GTL1-F-XmaI	5'-cccgggaatggagcaaggaggaggt-3'
GTL1-R-EcoRI	5'-cggaattcttactgaaccattgtcaagaaag-3'
AtCaM2-F	5'-atgttccagattacgctccgatggcggatcagctcacag-3'
AtCaM2-R	5'-tactagctagctggccagtcacttagccatcataaccttca-3'

Supplementary Table S2. Ramachandran plot statistics of GTL1N structure.

Ramachandran Plot Statistics	No. of residue	%
Residues in the most favored regions (A, B, L)	61	95.3[a]
Residues in additional allowed regions (a, b, l, p)	3	4.7
Residues in generously allowed regions (~a, ~b, ~l, ~p)	0	0.0
Residues in disallowed regions	0	0.0
	-----	------
Number of non-glycine and non-proline residues	64	100.0 %
Number of end-residues (excl. Gly and Pro)	2	
Number of glycine residues (shown as triangles)	2	
Number of proline residues	2	
Total number of residues	70	

[a]A good quality model is expected to have over 90% in the most favored regions.
